# Scalable manufacturing of multifunctional insect wing membrane via interfacial lase-and-peel strategy

**DOI:** 10.1126/sciadv.aea6934

**Published:** 2025-12-10

**Authors:** Jing Bian, Yuxing Ma, Hong Ling, Xiang Zhou, Lei Liu, Xingyu Yuan, Wenjing Li, Mengxin Gai, Brian Arianpour, Shaolei Wang, Yunlei Zhou, Junwen Zhong, YongAn Huang

**Affiliations:** ^1^ State Key Laboratory of Intelligent Manufacturing Equipment and Technology, Huazhong University of Science and Technology, Wuhan 430074, China.; ^2^ School of Microelectronics (School of Integrated Circuits), Nanjing University of Science and Technology, Nanjing 210014, China.; ^3^ Flexible Electronics Research Center, Huazhong University of Science and Technology, Wuhan 430074, China.; ^4^ Sensors and Systems Research Department, The 48th Research Institute of China Electronics Technology Group Corporation, Changsha 410111, China.; ^5^ College of Electronic and Optical Engineering and College of Flexible Electronics (Future Technology), Nanjing University of Posts and Telecommunications, Nanjing 210023, China.; ^6^ Department of Bioengineering, Henry Samueli School of Engineering and Applied Science, University of California Los Angeles, Los Angeles, CA 90095, USA.; ^7^ Department of Electromechanical Engineering and Centre for Artificial Intelligence and Robotics, University of Macau, Macau SAR, 999078, China.

## Abstract

Insect wings have unique, irregular nanopillars with multifaceted properties. One of the great challenges in broader applications of insect wing–inspired surfaces is to mass-produce insect-wing nanoarchitectures (IWNs) on ultrathin and durable substrates that can be integrated onto any planar/nonplanar surfaces. This study presents a simple method for producing IWNs on ultrathin polyimide (PI) films. An ultraviolet laser irradiates the PI-glass interface, generating gas that induces the nucleation, growth, and coalescence of nanobubbles, leading to the formation of nanopillars at the interface. Mechanical peeling then delaminates the PI film from glass along the middle of these nanopillars, creating IWNs on both PI film and glass. The artificial wing membrane (thickness < 500 nanometers) closely mimics natural dragonfly wings and can be seamlessly integrated onto contact lenses, demonstrating enhanced optical and bactericidal capabilities. This approach offers exceptional dimensional compatibility and high-throughput, potentially addressing scalability limitations that hinder broader applications of insect wing–inspired superfunctional surfaces.

## Teaser

A laser-and-peel method can mass-produce irregular, insect wing–inspired nanopillars on ultrathin, durable plastic substrates.

## INTRODUCTION

Numerous species in nature have exhibited extraordinary functionalities, such as plants (*1*, *2*) (liquid manipulation), insects (*3*–*7*) (optical manipulation/sensing), reptiles (*8*, *9*) (adhesion control), and marine life (*10*, *11*) (drag reduction). These properties, stemming from their distinctive nanoarchitectures, enable them to thrive under harsh environments. Particularly, insect wings have multifaceted functionalities that have drawn increasing attention in recent years (*3*, *7*, *12*). Irregular nanopillars (periodicity, ~100 nm; and height, 200 to 400 nm) on the cuticle of insect wings have been shown to enhance omnidirectional antireflection, bactericidal activity, and self-cleaning properties (*13*). Many insect species, including the dragonfly (*14*, *15*), glasswing butterfly (*12*, *16*), lacewing (*17*), damselfly (*18*), and planthopper (*19*), have similar surface topographies. This suggests that such nanostructures and their functionalities have been widely adopted by these species as critical survival adaptations (*20*).

The outstanding properties of insect-wing nanoarchitectures (IWNs) provide strong impetus to realize insect wing–inspired superfunctional surfaces (*21*). Unfortunately, due to the unique morphology features [i.e., high aspect ratios, random distributions, and variable cross section; (*20*)], it is still challenging to mass-produce IWNs with a high level of morphological and functional similarity by existing nanofabrication techniques. The template method, such as soft lithography, can produce diverse surface morphologies and partially mimic insect wing surfaces (*22*–*24*). However, the molding and demolding processes are challenging for nanostructures with high aspect ratios, and the achievable size remains constrained by the dimensions and cost of the replica template. By using etching, high–aspect-ratio nanofeatures can be fabricated on the surfaces of silicon/polymer with the similar bactericidal/antireflective performances as the natural dragonfly wings (*4*, *25*, *26*). Etching-based techniques, however, face challenges such as complex operations and low efficiency (*27*, *28*), often requiring numerous etching cycles due to limited etching depth per cycle (*25*), hindering rapid manufacturing. Recently, femtosecond-laser–induced nanostructures have been well-developed to realize the structural complexity of IWNs via point-by-point scanning (*13*). However, it still faces limitations for large-scale consistency due to the small spot area and nonuniform energy distribution of ultrafast lasers.

In addition to replicating the unique surface nanoarchitectures, it is also essential to mimic the macrofeatures of an insect wing, which is extremely lightweight and flexible, with a thickness of only 1 μm or even less (*20*). IWNs fabricated on ultrathin and durable films (e.g., substrates for ultrathin flexible electronics) that can be conformally attached on other planar/nonplanar surfaces [e.g., physical antimicrobial for orthopedic implants (*25*), sticker-type antireflective films for optoelectronic devices (*29*), and biomarker sensors for smart contact lenses (CLs) (*30*)]. As film thickness decreases, the conformality of a thin film substantially improves, potentially becoming self-adhesive as thicknesses below 1 μm (*31*), allowing seamless integration onto various planar and curved surfaces without additional adhesives (*32*). Therefore, fabricating a truly biomimetic insect wing membrane that combines both the “form” (ultrathin and durable) and the “essence” (nanopillared surface with superfunctionality) of natural archetypes can greatly expand the application scope of these bioinspired surfaces through conformal transfer printing onto nearly any object surface. However, traditional nanofabrication methods mainly rely on top-down surface treatments, which are highly sensitive to film flatness, depth processing accuracy, and substrate deformation control, making it difficult to apply to submicrometer-thick substrates. Moreover, compatibility issues arise with certain high-performance polymers, particularly those exhibiting high thermal resistance and chemical stability, such as polyimide (PI).

Here, rather than conventional surface treatments, we propose an avenue to mass-produce functional nanostructures on ultrathin and durable substrates by using laser-induced reactions at the interface. The manufacturing principle relies on the self-assembly of nanobubbles at the interface through laser confined ablation of a two-layered structure (PI-glass). Previous studies typically achieved complete interface separation between PI films and rigid glass substrates after laser irradiation, which has been used in flexible electronics and microchip assembly (*33*, *34*). However, we used incomplete interface separation (previously viewed as a failure in laser lift-off) to intentionally generate nanostructures at the interface. Subsequently, mechanical peeling then separates these interfacial nanopillars and creates irregular nanopillars instantly. The unique feature of physical coalescence of nanobubbles and mechanical fracture substantially simplifies the formation of irregular nanopillars and is fully compatible with submicron ultrathin films. The produced artificial wing is perfectly consistent with the natural wing not only in the micrometer-thick membrane, but in surface morphology and multifunctionality. The proposed technique is simple (with no need for a clean room/vacuum environment), chemical free, and mask free, and it can be easily scaled up using commercially available industrial laser-processing systems (table S3 presents a detailed comparison with existing nanofabrication methods, highlighting our advantages). Considering the artificial wing membrane given an exceptional conformability to other planar/nonplanar surfaces, this surface modification approach is universal and may be attractive for broad potential applications in medical devices and implants, biointegrated optical devices, and flexible/conformable electronics.

## RESULTS

### Fabrication capability of the lase-and-peel process

**Fig. 1.**
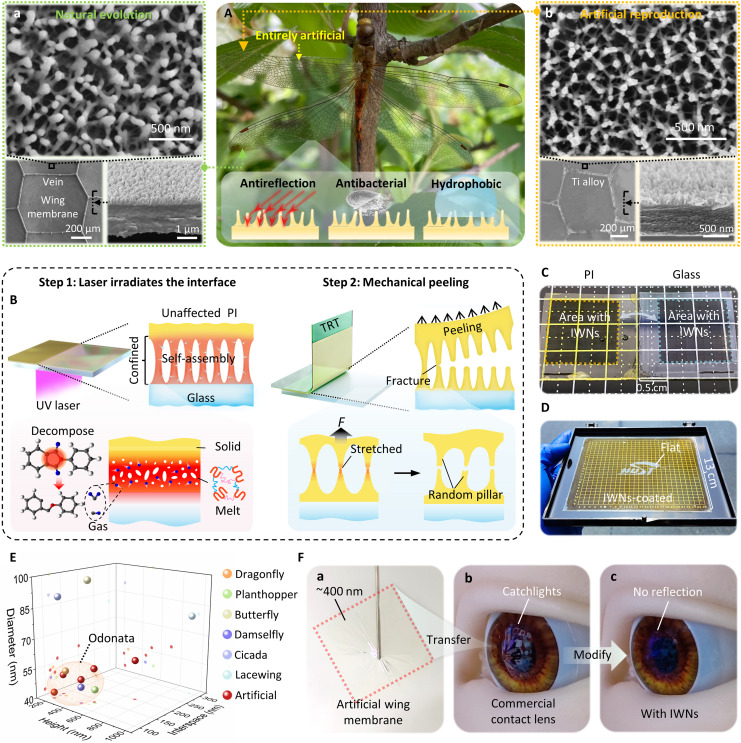
Fabrication capability of the lase-and-peel process. (**A**) The photo of a dragonfly in its natural environment, with an artificial wing (top left). Surface and cross-sectional SEM images show that the artificial wing [(A), b)] is perfectly consistent with the natural wing [(A), a]) not only in the macro morphology but also in surface nanostructures. The inset is the schematic diagram of the unique nanopillars, which are essential for omnidirectional antireflection, antibacterial, and hydrophobic behaviors. (**B**) The detailed procedure of the lase-and-peel process. (**C**) The photo of a PI film and a glass substrate with a square-shaped area of artificial insect-wing nanoarchitectures (IWNs) right after lase and peel. (**D**) The photo of a large-area IWN-coated PI film with a size larger than a human palm. (**E**) The typical dimensions of the natural IWNs and artificial IWNs produced in this study. (**F**) The photo of an artificial wing membrane with 400-nm thickness floating on the water [(F), a], which can be further transferred on a CL. The commercial CL exhibits the screen of the front smart phone [(F), b]. The modified CL barely reflects light in the same environment [(F), c].

As shown in Fig. 1A, in a dragonfly specimen, irregular nanopillars are found on the surface of a micrometer-thick wing membrane [see scanning electron microscopy (SEM) images in Fig. 1A(a)]. These sub-100-nm pillars exhibit irregular characteristics, including random height and distributions (e.g., a Gaussian height distribution with a variance of one-fifth the average height), a certain ratio of clustering (with some of nanopillars connected at the top), and cross-linked pedestals at the base. These features have been shown to be essential for achieving omnidirectional antireflection (*12*), antibacterial properties (*35*, *36*), and hydrophobic behaviors (*37*). With these functionalities, pairs of transparent, clean, and antireflective wings can substantially reduce the insect’s visibility and exposure. The lase-and-peel technique can reproduce these exquisite wing membranes with high fidelity. An entirely artificial wing has been fabricated (consisting of veins made of titanium alloy and an ultrathin artificial wing membrane) to be compared with a natural wing. As shown in Fig. 1A(b), the artificial wing aligns perfectly with the natural wing, not only in its submicrometer-thick membrane with delicate supporting veins but also in its surface nanostructures. As a result, the artificial wing is virtually indistinguishable in all respects.

Figure 1B presents the detailed procedure of the lase-and-peel process. Our approach first induces nanopillars at the near-interface of a two-layered structure (a PI film prepared on a glass substrate) and then generates nanopillars on the PI/glass surface through mechanical exfoliation. This process, which involves the physical coalescence of nanobubbles and mechanical fracturing in a massively parallel manner, shows great promise for scalable manufacturing. Specifically, a pulsed excimer laser with optimal fluence is used to repeatedly irradiate the PI-glass interface (step 1). During irradiation, only a very thin layer of PI (~300 nm) adjacent to the glass melts, generating tiny gas products from PI decomposition (*38*). The gas concentration at the near-interface can be gradually accumulated by increasing the irradiation number. The continuous release of gas then leads to the formation of high-density nanobubbles, which eventually coalesce to create a pillar-like morphology. Then, an adhesive carrier [e.g., thermal release tape (TRT)] can be bonded (or prefabricated) onto the PI film (step 2). Once the peeling force is loaded, these nanopillars will be stretched and broken so that artificial IWNs can form on both the PI and glass surface.

Figure 1C presents a picture of a PI film and a glass substrate with a square-shaped area of artificial IWNs, which are identical to each other as they came from the same sample. The area with IWNs is notably more antireflective and hydrophobic compared to the surrounding smooth surface. Notably, these IWNs, covering an area comparable to a natural wing, can be fabricated in just a few minutes using a common, nonindustrial experimental platform (see the whole fabrication process in movie S1). The proposed method exhibits excellent size scalability. As shown in Fig. 1D, a large-area IWN film, larger than a human palm, has been successfully fabricated. Only the central region is left uncovered by IWNs, allowing it to reflect light and display the “HUST” logo, while the surrounding area exhibits antireflective properties. Furthermore, the large-scale uniformity of the IWNs was confirmed by comparing antireflection performance and conducting microscopic morphological characterization across multiple distinct regions, as shown in fig. S1. Given that the typical energy consumption per unit area for our low-power excimer laser is only ~5 W/cm^2^ (e.g., ~100 mJ/cm^2^; irradiation number, 50; and frequency, 50 Hz), the present approach may obtain mass production (scanning efficiency > 100 cm^2^/s) by using a high-power industrial excimer laser system (power > 500 W, the calculation of scanning efficiency is presented in Supporting Information Text section A, along with figs. S2 and S3).

Figure 1E illustrates the basic dimensions (i.e., diameter, height, and interspace) of the nanopillars, which can be adjusted by modifying laser parameters and cover the size range of natural IWNs across various insect species. The size and morphology features of the nanostructures on insect wings are the outcome of a coevolutionary interplay among habit, environment, and functional demand. For instance, diurnal Odonata species inhabiting humid riparian zones use disordered nanopillars that effectively balance antireflective, antibacterial, and hydrophobicity properties. Notably, the artificial IWNs exhibit a high degree of structural similarity to natural Odonata wing nanostructures, with a 90% match across all morphological dimensions (see details in table S4). Additionally, numerous irregular features of natural IWNs have been successfully replicated, including random heights, clustered and free-standing distributions, and cross-linked pedestals at the base (see evidence in the enlarged SEM image in fig. S4). Moreover, because only ~300 nm of PI near the interface undergoes physical or chemical changes during laser irradiation, a damage-free lase-and-peel process remains achievable, even with PI thickness reduced to ~400 nm (thinner than a natural insect wing). Figure 1F(a) presents an image of a free-standing film (just 400 nm thick) after lase and peel, with no discernible ruptures. Due to its ultrathin structure and high transparency, the film is nearly invisible when floating on the water’s surface. This ultrathin film closely mimics the insect wing membrane, which can be further transferred onto other planer/curved objects to immediately endow them with fascinating bioinspired functionalities. For example, the biomimetic coating has been modified onto commercial CLs. As shown in Fig. 1F(b), the surface of the commercial CLs is highly reflective (clearly exhibiting the screen of a smart phone). In contrast, the eye with the modified CL barely reflects the light in the same environment [Fig. 1F(c)]. The modified CL maintains optical clarity while providing insect wing–inspired super-antireflective properties. The ability to mass-produce artificial wing membranes opens an avenue for direct and effective surface functional modification through direct heterogeneous integration.

### Formation of nanopillars at the interface

**Fig. 2.**
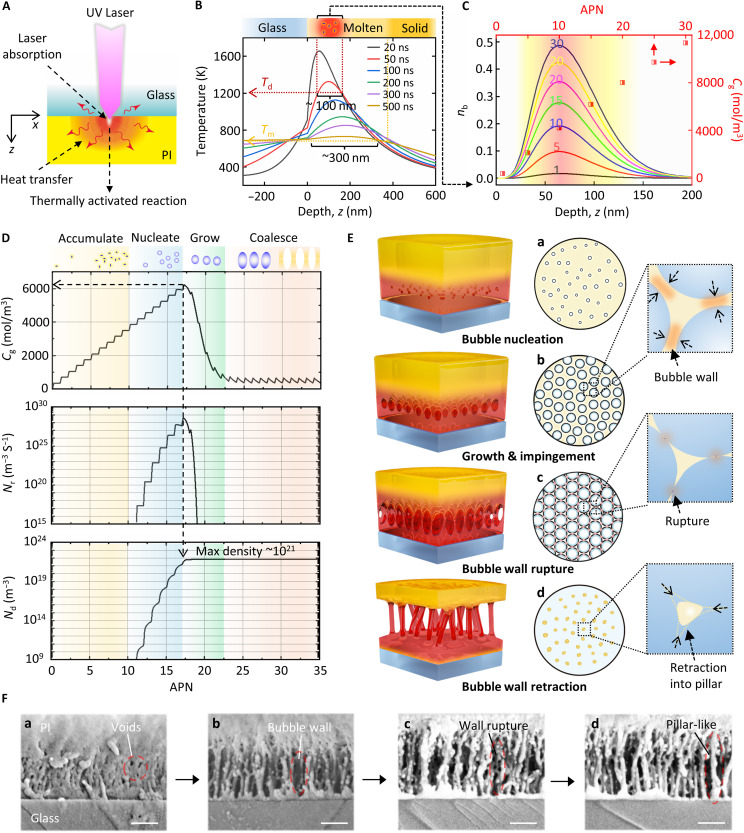
Process mechanism of gas-induced interface foaming. (**A**) Schematic diagram of the photothermal phenomenon caused by UV laser irradiation. (**B**) Temporal distributions of temperatures around the interface region under the laser irradiation of 100 mJ/cm^2^. (**C**) Distributions of broken bond fractions and the estimated gas concentration *C*_g_ under 30 sequential irradiations of 100 mJ/cm^2^. (**D**) The theoretical prediction of the gas concentration *C*_g_, the bubble nucleation rate *N*_r_, and the bubble nucleation density *N*_d_ under 35 sequential irradiations of 100 mJ/cm^2^. (**E**) Schematic diagram of the formation process of nanopillar-like interface nanoconnections. (**F**) A series of cross-sectional SEM images of different stages of the interface foaming process. [(F), a] Bubble nucleation (89 mJ/cm^2^, accumulated pulse number, APN = 30), [(F), b] bubble impingement (89 mJ/cm^2^, APN = 90), [(F), c] bubble wall rapture (89 mJ/cm^2^, APN = 150), and [(F), d] nanopillar formation (89 mJ/cm^2^, APN = 170). Scale bars, 200 nm.

The first key point of the fabrication of artificial IWNs is to form nanopillars at the interface, which requires two critical conditions: (i) The polymer must exhibit strong absorption of the ultraviolet (UV) laser (to form a nanoscale high-temperature region at the interface) and decomposes to release gas products; (ii) the rigid transparent substrate must transmit UV laser efficiently, remain stable at high temperatures, and have suitable thermal conductivity to regulate interfacial heat distribution; (iii) a robust initial interfacial adhesion between the polymer and the transparent substrate is essential to prevent premature delamination during processing. PI is particularly suitable for this process due to its molecular imide groups, which not only enable strong UV absorption (absorption coefficient > 10^4^ cm^−1^) but also facilitate molecular chain recombination and gas generation. The 308-nm excimer laser is selected because of its suitable light penetration depth (~100 nm), which matches the target nanostructure dimensions, its capacity to generate uniform laser spots enabling homogeneous large-area processing, and its ability to effectively transmit through most used rigid transparent substrates. The flat-top beam profile of an excimer laser allows us to only consider the laser-induced reactions in the vertical direction. As shown in Fig. 2A, the strong absorption of the UV laser (308 nm) causes high temperature at the interface, accompanied by several thermally activated physical and chemical reactions. A bulk photothermal model has been developed to simulate the temperature changes and the cleavage of the imide ring during laser irradiation (the detailed model is presented in Supporting Information Text section B) (*39*). Figure 2B presents the temperature distributions around the interface; when a moderate laser fluence is applied (e.g., 100 mJ/cm^2^, slightly over the ablation threshold of PI), the temperature of an extremely thin layer (~100 nm) of the PI film near the interface rapidly exceeds the decomposition temperature of PI (~1100 K) within tens of nanoseconds. Around the decomposition region (~ 300 nm in thickness), the high temperature causes the PI to melt (~700 K) within a few hundred nanoseconds, creating a unique situation where gas products from PI decomposition can reshape the molten PI. If the laser fluence is too high (>120 mJ/cm^2^) or too low (<60 mJ/cm^2^), it results in carbonization (*40*) or nondecomposition of interfacial PI (*41*), respectively, rather than forming nanopillars.

The accumulated pulse number (APN) is another crucial process parameter. Figure 2C illustrates the simulated distributions of broken chemical bonds, showing that, as the APN increases (from 1 to 30), the fraction of broken bonds, *n*_b_, rises with the number of irradiations. These broken PI bonds generate gas products. The photothermal model can predict the amount of gas generated at the interface, with its accuracy verified in our previous research (*42*). The amount of gas products *n*_g_ has been proven to be proportional to the total broken bonds *N*_b_ [$N_{b}=\int_{0}^{\infty }n_{b}\left(z\right)\mathrm{dz}$], i.e., *n*_g_ (nanomoles per square centimeter) = 1.5 *N*_b_ (*39*). A high gas concentration will be formed in the range of about 50 to 100 nm away from the interface (red region in Fig. 2C). To simplify the analysis, the gas diffusion process is neglected because of the high-temperature gradient near the interface region. We focus on the 50-nm-thick region with the highest temperature and assume that the gas concentration is uniformly distributed within this region. The average concentration of gas *C*_g_ (red squares in Fig. 2C, presume *C*_g_ = *n*_g_/50 nm) gradually increases with the APN. This nanoscale gas accumulation within the molten polymer is a unique phenomenon, attributed to the strong UV absorption of PI, efficient heat conduction of the glass, and the confined interfacial environment. On the one hand, it ensures a highly localized affected region, matching the nanoscale of natural IWNs. On the other hand, gradual gas accumulation prevents mechanical damage to the ultrathin upper PI film, as only a small amount of gas will be produced with each irradiation.

With the increase of gas concentration, gas supersaturation will lead to bubble nucleation, followed by bubble growth and impingement. This process is similar to the gas foaming technology for the fabrication of open-cell polymeric foams (*43*). However, the foaming at the interface is confined to an extremely thin layer, resulting in the planar arrangement of nanobubbles. In this study, accurately simulating the polymer foaming process is challenging due to the extremely small scale, nonuniform gas concentration, transient temperature changes, and unclear polymer properties. Therefore, this study focuses on establishing the relationship between laser parameters and interface cavitation evolution rather than simulating every detail of bubble coalescence. A simplified physical model combining the classical nucleation theory, a bubble growth model, and the photothermal model is developed to qualitatively describe the nucleation and growth of nanobubbles, which assumes that the bubble nucleation rate *J*(*t*) and the bubble growth rate *dR*/*dt* (*R* is the bubble radius) depend on *C*_g_ (*t*) at time *t* (the detailed model in Supporting Information Text section C, along with fig. S5) (*44*). One can observe from Fig. 2D that the interface foaming can be divided into four stages. The first few irradiations (APN < 10) do not induce bubble nucleation, only leading to the increase of *C*_g_ (stage 0). Once the *C*_g_ reaches a high level, the nucleation rate *N*_r_ will substantially increase. Just a few irradiations could rapidly increase the nucleation density *N*_d_ to an ultrahigh value of 10^21^/m^3^ (stage 1). Subsequently, the high-density bubbles enter a rapid growth period (stage 2) as *C*_g_ remains at a high level, thereby rapidly consuming previously accumulated gas in the polymer. *C*_g_ quickly drops to a low level, inhibiting further nucleation. Afterward, *C*_g_ stabilizes at a low level, so all newly generated gas from subsequent irradiations is used exclusively for bubble growth, promoting bubble coalescence (stage 3).

Figure 2 (E and F) presents a series of schematic diagrams and SEM cross-sectional images to illustrate the interface foaming process. Initially, no cavitation could be found (fig. S6). Upon bubble nucleation [Fig. 2E(a)], small nanovoids appear around the PI-glass interface [refer to nanovoids in Fig. 2F(a)]. As high-density nanobubbles grow, their coalescence becomes inevitable (*45*). Figure 2E(b) illustrates that, as the high-density nanobubbles grow, they begin to “sense” each other, gradually transforming into ellipsoidal shapes. The polymer layer separating them is compressed and progressively thins (*46*). Accordingly, as shown in Fig. 2F(b), the bubble wall structure separating bubbles has been observed. The molten polymer occupying the space between the bubbles undergoes extension (*43*, *46*), allowing it to reach specific rupture criteria. Figure 2E(c) captures the moment when the bubble wall ruptures, with several holes forming in the middle of the wall, as shown in in Fig. 2F(c). Subsequently, as depicted in Fig. 2E(d), once the rupture occurs, the thin wall separating the bubbles retracts, driven by surface tension, which acts against viscous forces to minimize the interface area. Last, an open-cell morphology gradually forms, with the polymer confined to cell struts (i.e., spacer regions between adjacent bubbles) (*43*), as seen in the pillar-like nanostructures in Fig. 2F(d). In summary, by precisely generating gas within the molten polymer, an open-cell morphology is achieved via physical bubble coalescence, efficiently forming nanopillars at the interface. We have provided additional cross-sectional SEM images that illustrate the entire evolution, as shown in fig. S7.

### Path-selected interface separation

**Fig. 3.**
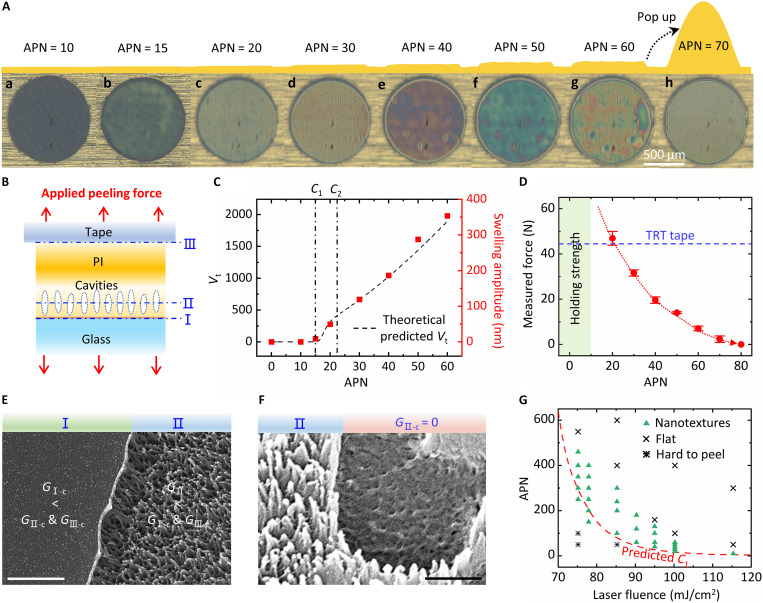
Path-selected interface separation during the mechanical peeling. (**A**) Optical photographs and surface profiles of the same region under increasing irradiation number, APN = 10 to 70, and 100 mJ/cm^2^. (**B**) Schematic illustration of the path-selected mechanical peeling process. (**C**) The total bubble volume *V*_t_ obtained through theoretical calculations varies with the irradiation number, which is consistent with the trend of the measured swelling height shown in the right. (**D**) The measured interface bonding strength of PI-glass samples under different irradiations number. The dotted line is the measured adhesive force of the TRT tape used in the experiment. (**E** and **F**) The SEM observations of the peeled PI film after different irradiation number present direct evidence of three possible separation paths. (E) APN = 10 and (F) APN = 70. Scale bars, 500 nm. (**G**) The appropriate laser parameters required for surface nanostructures. The dotted line is the predicted critical irradiation number *C*_1_ as the criterion for determining whether the PI film can be mechanically peeled.

Figure 3A and fig. S8 display optical images and surface profiles of the same region under varying numbers of laser irradiations (at ~100 mJ/cm^2^). Correspondingly, the interface bonding strength at different stages has been measured (see fig. S9 for details). No visual changes or swelling are observed after just a few irradiations [APN = 10, Fig. 3A(a)], indicating that nucleation has not yet occurred (stage 0). As shown in Fig. 3D, at this stage, the interface bonding strength remains high (beyond measurable limits). The critical irradiation number *C*_1_ for massive nucleation is predicted to be ~15. The optical observation [Fig. 3A(b)] shows a transparency change in the PI-glass sample and profile measurement confirms a very small bulge (~9 nm) at APN = 15, indicating the formation of numerous tiny bubbles (stage 1). Subsequently, these nanobubbles grow rapidly (stage 2). The critical irradiation number *C*_2_ (indicating the end of rapid growth) is predicted to be ~22. Experiments demonstrate that, at APN = 20, the swelling height increases to ~50 nm, reflecting a period of rapid nanobubble growth. In stage 3 (APN = 30 to 60), the gas concentration stabilizes, maintaining constant cavitation growth. Experiments also confirm a linear relationship between PI swelling height and irradiation number, with interface bonding strength gradually decreasing at this stage (Fig. 3D). Figure 3C shows calculated nanobubble volume *V*_t_, which is consistent with the trend of the measured swelling height of the PI surface. Additional experiments of the evolution of interface cavitation under different laser fluences are provided in figs. S10 and S11. The above results indicate that the theoretical models can predict the key stages of interfacial cavitation, especially the critical point *C*_1_. After this stage, many grown nanobubbles appear at the interface, which will substantially weaken the PI-glass interface, thereby ensuring the feasibility of mechanical peeling.

The peeling process can be achieved using an adhesive tape that is compatible with roll-to-roll methods for large-scale applications. There are three possible separation interfaces during mechanical peeling: interface I (PI/glass), interface II (inside the cavitation region), and interface III (adhesive tape/PI surface), as shown in fig. S12. According to Griffith’s criterion, the interface with a higher *G*/*G*_c_ (*G* is the energy release rate for delamination and *G*_c_ is the critical energy release rate) is more likely to delaminate (*47*). To prepare IWNs, interface II must delaminate first, requiring *G*_II-c_ < min(*G*_I-c_, *G*_III-c_). Here, *G*_I-c_ depends on the adhesion strength between the PI and glass, *G*_III-c_ depends on the stickiness of the tape, and *G*_II-c_ depends on the cohesive strength of the PI material. For an untreated sample, *G*_II-c_ > *G*_I-c_ > *G*_III-c_. When cavitations exist inside the polymer, its cohesive strength decreases with increasing porosity (*48*), substantially reducing *G*_II-c_. The key to manufacture IWNs lies in using the laser-induced interface cavitation to reduce the *G*_II-c_ to a sufficiently low level to meet the condition: *G*_II-c_ < *G*_III-c_.

Therefore, the separation path may not be fixed and depends on the degree of interface foaming. For a fixed laser fluence (e.g., 100 mJ/cm^2^), only a certain range of the APN (e.g., APN = 20 to 60) could produce surface nanostructures. With insufficient foaming, even if mechanical peeling is successful, separation likely occurs at the exact PI-glass interface (*G*_I-c_ < *G*_II-c_, denoted as path-I in Fig. 3B), resulting in a smooth surface. Conversely, with excessive irradiations, separation occurs due to the loss of interfacial nanoconnections rather than mechanical fracture (i.e., *G*_II-c_ = 0), also yielding a smooth surface. Figure 3 (E and F) presents the direct evidence of all three possible separation paths. When the peeling is performed too early (i.e., APN = 10), competition between path-I and path-II could occur, as the left part in Fig. 3E is peeled along path-I, while the right part is peeled along path-II. If the peeling is conducted too late (i.e., APN = 70), as seen in Fig. 3F, then the left part retains nanotextures, while the right part appears smooth. To obtain IWNs, the exact condition should be 0 < *G*_II-c_ < min(*G*_III-c_, *G*_I-c_).

Figure 3G presents a parametric study to determine the optimal timing for mechanical peeling. The process window for achieving nanostructures varies with the applied laser fluence. This variation arises from gas generation during PI thermal decomposition, which follows an Arrhenius-type kinetics (*39*), with reaction rate exponentially dependent on temperature. Consequently, even a slight increase in laser fluence (effectively raising interface temperature) can greatly increase gas production, thereby resulting in pronounced differences in the process window. When interface cavitation enters the rapid growth stage, PI porosity increases, substantially weakening *G*_II-c_. We use *C*_1_ as the criterion to predict whether the PI film can be mechanically peeled. The theoretical predictions are generally consistent with the experimental results. We further evaluated the adhesion force of the TRT tape, which is comparable to the interface bonding force of the peelable PI-glass sample (*F*_laser_ = 100 mJ/cm^2^, APN = 20), proving that *G*_II-c_ < *G*_III-c_ could be satisfied under this condition. Additionally, experiments using tapes of different viscosities (fig. S13) confirm that high adhesive tapes can effectively expand the process window, as it becomes easier to satisfy: *G*_II-c_ < *G*_III-c_. Additionally, the experimental results (fig. S14) further demonstrate that the mechanical peeling process has no limitations on film thickness. Even 400-nm-thick PI films can be peeled intact and without damage using TRT. This effective peeling can be attributed to two factors: First, the PI surface remains smooth after cavitation, enabling seamless adhesion between TRT and PI. Second, the substantially reduced bonding strength between the PI and glass minimizes stress during mechanical peeling.

It is likely that the interface nanostructures first undergo some plastic deformation during stretching before breaking in the middle, which is typically the weakest point in the connection. As shown in fig. S15, comparison of the nanostructures on the peeled PI film and glass substrate (from the same region before peeling) confirms that these structures exhibit similar morphological features. This finding provides evidence that these nanoconnections tend to break in the middle. Additionally, the combined height of the nanostructures on the PI film surface and the glass surface exceeds that of the interface nanopillars (as estimated by the swelling amplitude of the PI film before peeling), suggesting that some plastic deformation occurs during the peeling process.

### Regulation of morphological features

**Fig. 4.**
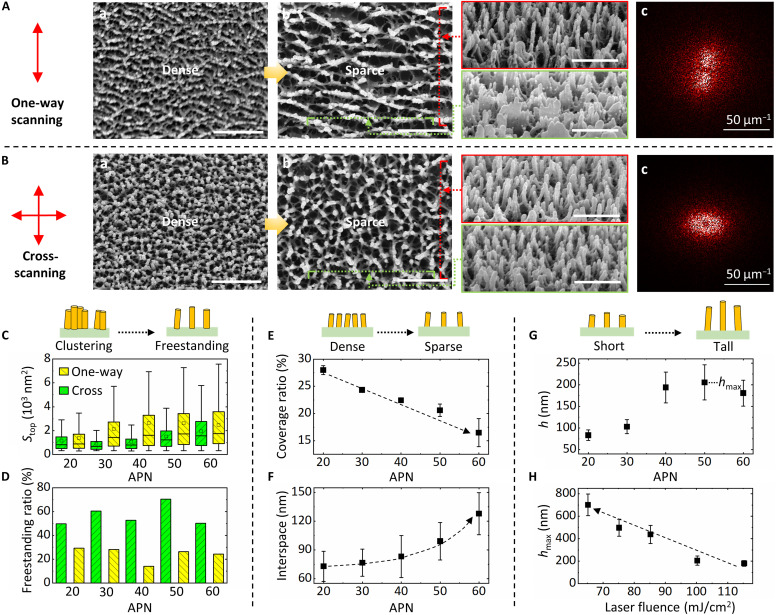
Regulation of morphological features of nanostructures through control of process parameters. (**A**) SEM images of surface nanostructures. [(A), a] One-way scanning, *F*_laser_ = 80 mJ/cm^2^, and APN = 100. [(A), b] Top-view and side-view SEM images of surface nanostructures, one-way scanning, *F*_laser_ = 80 mJ/cm^2^, and APN = 460. [(A), c] 2D fast Fourier transform (2D-FFT) of [(A), b]. Scale bars, 500 nm. (**B**) SEM images of surface nanostructures. [(B), a] Cross scanning, *F*_laser_ = 80 mJ/cm^2^, and APN = 55(*x*) + 55(*y*). [(B), b] Top-view and side-view SEM images of surface nanostructures, cross scanning, *F*_laser_ = 80 mJ/cm^2^, and APN = 180(*x*) + 180(*y*). [(B), c] 2D-FFT of [(B), b]. Scale bars, 500 nm. (**C**) Top-side area *S*_top_ of nanostructures as a function of APN. (**D**) Freestanding ratio of nanostructures as a function of APN. (**E**) Coverage ratio of nanopillars as a function of APN. (**F**) Interspace of nanopillars as a function of APN. (**G**) The average heights of nanopillars as a function of APN; *F*_laser_ is fixed at 100 mJ/cm^2^. (**H**) Achievable maximum heights of nanopillars as a function of applied laser fluence.

The distribution pattern, density, height (aspect ratio), and some unique morphological features of the fabricated nanostructures can be precisely controlled by adjusting laser scanning parameters, such as laser fluence, APN, and scanning strategy. First, the distribution form of nanostructures can be transferred from freestanding nanopillars to cluster-like nanosheets. Nanostructures prepared by one-way scanning exhibit an anisotropic distribution (Fig. 4A), forming clusters perpendicular to the scanning direction in the shape of parallel nanosheets. Side-section images taken from two angles reveal that these nanosheets resemble nanomountains. The corresponding two-dimensional fast Fourier transform (2D-FFT) image [Fig. 4A(c)] further confirms the unidirectional periodicity of nanosheets. In contrast, when cross-scanning is applied, as shown in Fig. 4B, the resulting nanostructures take the form of nanopillars. Side-section images and the 2D-FFT image [Fig. 4B(c)] confirm an isotropic, uniform distribution of these nanopillars. This phenomenon is further validated by nanostructures created with different scanning strategies on the same sample, as demonstrated in fig. S16. We assume that this phenomenon could be related to the laser-induced periodic surface structure. The laser has polarization due to the energy attenuator, which may lead to periodic modulation perpendicular to the laser polarization (*13*). By using cross-scanning, the equivalent contribution of laser energy could be achieved to form uniformly distributed nanostructures.

Morphological analysis has been performed by particle analysis of top-view SEM images in fig. S17 (see details in Supporting Information Text section D, along with fig. S18) to obtain the nanostructure density and whether they are clustering or freestanding. As shown in Fig. 4C, nanostructures prepared by cross-scanning have smaller topside area *S*_top_. Additionally, more than half of the nanostructures pass circularity filtration (Fig. 4D), further indicating that freestanding nanopillars can be effectively fabricated using cross-scanning. The nanopillar radius, estimated from *S*_top_, is measured in the range of 15 to 30 nm. As shown in Fig. 4E, the coverage ratio of nanostructures decreases progressively as the APN increases, indicating a gradual reduction in density. The spacing between nanopillars (indicating density) is estimated to range from 60 to 150 nm (Fig. 4F). These findings suggest that nanobubbles coalesce progressively with increasing APN. The average height of nanopillars is estimated from side-view SEM images. As shown in Fig. 4G, the height of nanopillars exhibits a trend of first increasing and then decreasing with APN, as evidenced by representative SEM images in fig. S19. This increase in height results from the vertical growth of nanocavities. In the final stage, the sparse and slender nanopillars tend to cluster, leading to a reduction in height. The obtained maximum height is found to be related to the applied laser fluence (representative SEM images shown in fig. S20). As shown in Fig. 4H, taller nanostructures could be achieved under lower laser fluence conditions. This phenomenon may be related to the rheological behavior of viscoelastic polymer melts. At lower fluences, the reduced gas production allows for a more gradual and stable thinning process of the liquid bridge (the evolution of interface nanostructures). In contrast, high laser fluences produce more gas, inducing rapid stretching of the liquid bridge and increasing the likelihood of premature rupture, thereby limiting the growth of interfacial nanostructures.

To summarize concisely, the optimal laser parameters for fabricating artificial IWNs are cross-scanning (to achieve pillar-like shapes), a high APN (to create larger interspaces), and a low laser fluence (to attain a high aspect ratio). Notably, several key features of natural IWNs have been successfully reproduced. First, the distribution, morphological features, and height of the prepared nanopillars exhibit random characteristics, due to the stochastic nature of the nucleation and pillar-fracture processes. For example, our artificial IWNs also have a Gaussian height distribution with a variance of σ_h_ = 40 to 80 nm [σ_h_ = ~40 nm for natural dragonflies; (*14*)], and their freestanding ratio is 50 to 70% [50 to 60% for natural dragonflies; (*14*)]. The clustering of nanopillars may be attributed to the minimization of surface energy, as the cluster structure exhibits greater stability compared to independent nanopillars and has a smaller total surface area. Second, just like natural IWNs (see SEM image in fig. S21), tree-root–like pedestals have been observed at the base of the nanopillars. Due to the planar arrangement of nanobubbles, the squeezing of the spacer region of several adjacent nanobubbles bottom (or top) is not obvious. The “tree-root–like” pedestal could be the residue of the ruptured bubble wall after retraction. Additionally, with one-way scanning, the resulting nanosheets resemble the surface protrusions on natural green lacewing wings (*49*), as shown in fig. S22.

### Multifunctional surface beyond the natural prototype

**Fig. 5.**
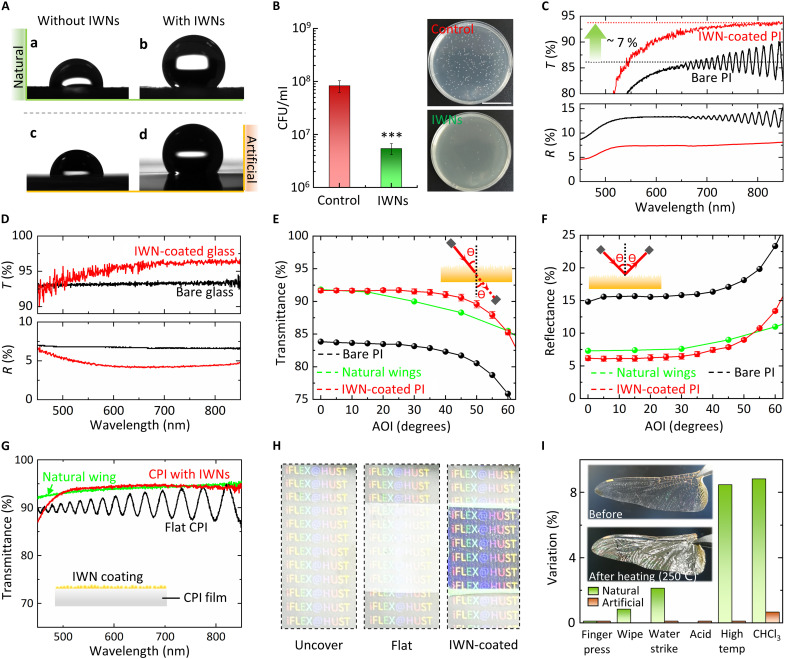
Demonstrations of multifunctional surface properties of artificial IWNs. (**A**) Hydrophobicity of a single water droplet on the dragonfly wing surface and PI surface without and with IWN coating. (**B**) Counts of viable *S. aureus* after being incubated with 0.8 cm–by–0.8 cm IWN-coated PI film and a bare PI film (as control) for 24 hours. Data are presented as means ± SD; *n* ≥ 3; biological replicate; and statistical significance is denoted by ***P < 0.001. The right part is the representative results of plate counts. (**C**) Transmission and reflectance spectra of an IWN-coated PI film and a bare PI film (~12 μm, commercial). (**D**) Transmission and reflectance spectra of an IWN-coated glass and a bare glass. (**E** and **F**) Optical transmittance and reflectance of an IWN-coated PI film and a bare PI film at various incidence angles. AOI, angle of incidence. (**G**) Transmission spectra of IWN-coated CPI films, which are compared with a flat colorless PI (CPI) film (~5 μm, homemade) and a natural dragonfly wing. (**H**) Demonstrations of antireflection effect of the IWN-coated CPI film. (**I**) Variations of transmittance of IWN-coated PI films and dragonfly wings after various mechanical stability and chemical stability tests; inset images show a dragonfly wing before and after the durability tests.

Due to the high structural similarity in all aspects, artificial IWNs have similar functionalities to natural ones. As shown in Fig. 5A [(a) and (b)], the natural IWNs demonstrate substantially enhanced surface hydrophobicity [water contact angles (WCAs), from 93° to 134°]. Besides, the self-cleaning abilities of insect wings are transient (*50*). Prolonged contact with water will cause natural wings to lose their hydrophobicity (fig. S23A). The increase in static water pressure from large water droplets promotes penetration of the liquid into the underlying nanostructures. Thus, IWNs focus on immediately repelling small water droplets to ensure flight performance, rather than providing long-term resistance to large volumes of water. Artificial IWNs exhibit similar hydrophobic characteristics. As shown in Fig. 5A [(c) and (d)], the WCAs of the smooth PI and IWN-coated PI are 85° and 133°, respectively. The artificial IWNs also exhibited a gradual decrease in hydrophobicity over time (fig. S23B).

Unlike the orderly arrangement of cicada wing nanopillars (*51*), the irregular and randomly distributed nanopillars can exert a greater shearing force on adhered bacteria when the bacteria attempt to move (*52*). We chose Gram-positive bacteria, which have harder cell walls (thus, more challenging to kill), as the test subjects. The bactericidal efficacy of artificial IWNs has been confirmed by the cell-viability plate counts (the detailed method is presented in Supporting Information Text section E, along with fig. S24). By covering the bacterial solution [2 ml, 10^5^ colony-forming units (CFU)/ml] with IWN-coated PI films, compared with planar PI films as controls, over 95% antibacterial rate of *Staphylococcus aureus* is determined after 24 hours of incubation from three independent experiments (Fig. 5B, more detailed results of plate counts are shown in fig. S25).

The antireflective ability of artificial IWNs has been thoroughly verified. Nonpolarized light was used in our measurements, which is a common method for evaluating isotropic nanostructures. As shown in Fig. 5C, the PI film with artificial IWNs exhibits a ~7% increase in transmittance compared to a flat film. The periodic oscillations in the transmission and reflection spectra of smooth PI films arise from interference between light reflected from the top and bottom surfaces, with the period closely related to the film thickness. In samples with surface nanostructures, the oscillation is negligible due to strong attenuation of the top-surface reflection. For an IWN-coated silica glass, its transmittance is also enhanced ~3.7% (Fig. 5D). It is worth highlighting that the antireflective performance of artificial IWNs is at a comparable level to that of the current state-of-the-art single-sided antireflective nanotechnologies both for PI and glass substrates (*53*–*55*). The haze of the IWN-coated glass is below 1% (fig. S26), demonstrating the high clarity. Moreover, angle-resolved reflectance and transmittance spectra were recorded at different wavelengths (from 550 to 1100 nm), as presented in Fig. 5 (E and F, respectively). The overall transmission of the IWN-coated PI film remains nearly unchanged, with only a slight decrease at larger incident angles, indicating an omnidirectional antireflective property. The IWN-coated PI film has similar antireflection performance as the natural wing in the yellow-red waveband [the green points in Fig. 5 (E and F), obtained from the literature (*26*)].

We conducted a detailed study of nanopillar profiles on its antireflection performance (details in Supporting Information Text section F, along with figs. S27 to S29). The artificial IWNs have a suitable period and height, and their random height distribution and bottom pedestal enable a gradual change in the effective refractive index at the interface (*12*). Building on this, an ultrathin IWN coating (400 nm thick) can be directly applied to a common transparent polymer film [e.g., colorless PI (CPI)] to achieve antireflective properties with minimal impact on full-spectrum transparency. As shown in Fig. 5G, the IWN-coated CPI film performs comparably to a natural dragonfly wing across most of the visible spectrum, while the transmittance of a flat film is only about 90%. Figure 5H illustrates the effects of the IWN-coated CPI film under strong light compared to a flat film. Through the flat film, it is difficult to clearly see the colored letters below, while the IWN-coated film maintains clarity and color fidelity. This transparent IWN-coated film can serve as a cover for optoelectronic devices; for instance, it notably reduces the unwanted reflection on the crease of foldable displays, as demonstrated in fig. S30.

The mechanical and chemical stability of IWNs is crucial for practical applications and was assessed by measuring transmittance changes after testing (Fig. 5I). For mechanical stability, artificial IWNs can withstand external forces without performance degradation (see movie S2), including finger pressing (~150*g* force, applied 20 times), wiping with a screen cloth (20*g* additional force, 10 times), and water impact (2 liters of water from a height of 50 cm). In terms of chemical stability, artificial IWNs demonstrate excellent thermal resistance (250°C for 4 hours) and tolerance to organic solvents. The natural components of IWNs (primarily wax) melt at high temperatures and dissolve in trichloromethane (CHCl_3_), which substantially increases reflection. Inset images of Fig. 5I show a dragonfly wing before and after a durability test. An obvious increase in the optical reflection is visually apparent. In contrast, artificial IWNs show no visible changes (fig. S31). Analogous to nanoimprinting, laser-induced interface cavitation enables morphological reshaping of the polymer through a heating-shaping-cooling process. The shape transformation is based on a physical mechanism that does not substantially alter the polymer’s mechanical/chemical properties, and the resulting nanostructures are permanently “frozen” in place upon cooling, thereby achieving robust structural stabilization.

### Insect wing–inspired CLs

**Fig. 6.**
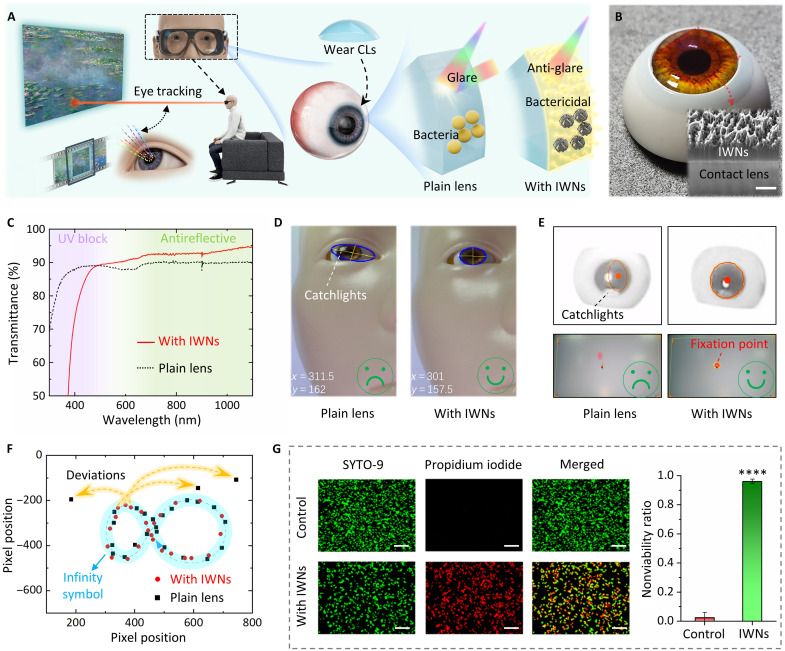
Demonstration of insect wing–inspired multifunctional CLs. (**A**) Schematic illustration of the eye-tracking technology used in mixed reality applications. The illustrations were created entirely by the authors. The 3D models were constructed using C4D 2024.2 and rendered with Redshift 3.5.24. (**B**) The photo of insect wing–inspired CLs. The inset is the cross-sectional SEM image showing the ultrathin film can conform to the surface of the CL. Scale bar, 500 nm (**C**) The transmittance test results of CLs before and after modification. (**D**) The test results of anti-interference capability of plain/modified CLs for visible-light tracking mode. (**E**) The test results of anti-interference capability of plain/modified CLs for near-infrared-light tracking mode. (**F**) The infinity symbol calligraphed by eye movement under strong light interference using plain/modified CLs. (**G**) Fluorescent micrographs of a negative control and nanostructured surface after 24 hours of incubation in a culture of *S. aureus*. Scale bars, 10 μm. Adherent bacteria are labeled with cell-permeable nucleic acid markers SYTO 9 (green) and propidium iodide (red). The bacterial antiadhesion ability is measured as the ratio of dead/total cell coverage. Data are presented as means ± SD; *n* ≥ 3; biological replicate; and statistical significance is denoted by ****P < 0.001.

As illustrated in Fig. 6A, eye tracking is vital for enabling efficient interactions with virtual environments (*56*). Current commercial eye-tracking systems mainly rely on pupil-center corneal reflection using visible or near-infrared light sources. However, traditional CLs can impede eye tracking due to environmental light interference (*57*). Additionally, prolonged use of CLs without proper ocular hygiene increases the risk of bacterial adhesion and subsequent ocular infections (*58*). Thus, there is a pressing need for CLs with improved anti-glare and antibacterial properties. The fabricated artificial wing membrane, with its submicron thickness, can be easily integrated onto various flat or curved surfaces using a promising heterogeneous integration technique (e.g., water transfer printing). Experimental results have confirmed that the release of the PI film from the TRT is highly sensitive to film thickness (fig. S32), making it challenging to obtain freestanding submicron-thick films. To address this issue, a thick polyvinyl alcohol (PVA) sacrificial layer is introduced as an intermediate contact medium between the PI film and the TRT, enabling complete detachment from the TRT and lastly yielding free-standing films after removing PVA by hot water. The artificial wing membrane naturally floats at the water-air interface; after being lifted from the surface of water, it naturally conforms to the target with the assistance of surface tension, adhering tightly as the water evaporates with no adhesive required. As shown in fig. S33, it even conforms to surface wrinkles with a bending radius of less than 500 nm, demonstrating exceptional conformability. Building on this, insect wing–inspired CLs have been developed with enhanced optical, bactericidal, and UV-blocking capabilities. As shown in Fig. 6B, due to its intimate adhesion and ultrathin thickness of ~400 nm, the biomimetic coating is nearly invisible, preserving optical clarity (see more images in fig. S34).

The transmittance of CLs before and after modification (Fig. 6C) demonstrates that the modified CL can almost completely block UV light while enhancing transmittance in the visible and near-infrared wavelength range. This UV blocking ability is not attributable to scattering effects from the nanostructures (*30*, *59*) (because their period is sufficiently small) but rather arises from the intrinsic strong UV absorption of the PI material. Using a professional eye-tracking equipment, the anti-interference capability of modified CLs was tested (the test method shown in fig. S35). As illustrated in Fig. 6D, for visible-light tracking mode, tracking failure occurs when an external light source creates catchlights near the pupil. As contrast, for the modified CL, no such catchlights are observed under identical circumstances. Furthermore, as shown in Fig. 6E, for near-infrared-light tracking mode, the interfering light can easily induce fluctuations in fixation points that impede accurate recognition. In contrast, the modified CL maintains a stable fixation point under similar interference. The infinity symbol “∞” was calligraphed by eye movement under strong light interference, as shown in Fig. 6F and movie S3. With plain CLs, accurate eye drawing of the symbol is difficult, as obvious deviations in gaze points occur at specific locations due to interference. Using the modified CLs, the infinity symbol is well drawn by the continuous eye tracking, exhibiting strong anti-interference performance in complex environments.

Figure 6G presents the bacterial antiadhesion ability of the biomimetic coating along with glass slides as negative controls. After incubation times of 24 hours, bacterial viability of *S. aureus* (the main cause of bacterial eye infection) was measured using a live/dead fluorescence assay consisting of cell-permeant nucleic stains SYTO-9 and propidium iodide. The propidium iodide only stains the nonviable bacteria with damaged cell membranes with fluorescent red. There was a large amount of green fluorescence with very little red fluorescence on the control surface. In contrast, a remarkable increase in red fluorescence and substantially decreased green fluorescence on biomimetic surfaces showed notable antiadhesion and bactericidal effects. Fluorescence quantification analysis (measured as the ratio of dead/total cell coverage) revealed the killing efficacy of >96% against *S. aureus*, demonstrating notable advantages over traditional CLs.

## DISCUSSION

In summary, a scalable fabrication technique is presented that enables cost-effective, large-scale, and high-fidelity reproduction of insect wing–inspired irregular nanopillars on ultrathin PI substrates. Theoretical and experimental studies reveal that the gas products could reshape the surrounding molten PI during laser irradiation, initially forming nanopillars at the interface. Mechanical peeling then fractures these interfacial nanopillars and creates irregular nanopillars on both the PI film and glass. The morphology of nanopillars can be precisely adjusted through the laser parameters, achieving the ultrahigh structural similarity. The artificial IWNs exhibit surface functionalities comparable to natural wings, demonstrated in hydrophobicity, antibacterial properties, and antireflection. This technology can efficiently reproduce the insect wing membranes, allowing the artificial membrane to conform seamlessly to a wide range of shapes and surfaces under hydraulic pressure. An insect wing–inspired CL with enhanced optical (anti-interference ability for eye tracking) and bactericidal (killing efficacy of >96% against attached *S. aureus*) capabilities has been demonstrated. This technique may enable mass production through the utilization of an industrial excimer laser system with potential efficiency exceeding 100 cm^2^/s.

In the future, we aim to extend IWN fabrication to a wider range of polymer materials and transparent substrates. Optimizing process parameters and procedures is essential to achieve diverse nanostructure dimensions and morphologies. By tuning key parameters, such as laser wavelength, polarization, scanning path, and mechanical peeling, the size, anisotropy, and orientation of nanostructures can be precisely controlled. Furthermore, adopting laser interference patterns or prepatterned nanostructured transparent substrates may enable hierarchical nano-on-micro multiscale architectures. In addition, further exploration of the capacity to double-sided functionalization is feasible. Strategies such as constructing a “glass-PI-glass” sandwich structure or prefabricating two single-sided functionalized thin films followed by seamless bonding are expected to further improve optical or antibacterial performance. Recently, breakthroughs in drop-printing and soap-transfer technology have enabled the nondestructive transfer of ultrathin devices onto complex 3D surfaces (*60*, *61*), broadening the use of artificial wing membranes. Integrating functional devices on the membrane backside allows dual functions (surface modification and real-time sensing) to be achieved simultaneously, paving the way for smart biointegrated systems. A promising future direction is “cyborg insects” (*62*), where natural wings are replaced with artificial membranes embedded with devices for sensing, wireless communication, and controlled flight, while maintaining natural flight capability.

## MATERIALS AND METHODS

### Preparation of PI-glass structure

The preparation of the PI-glass structure began with spin-coating adhesive of the nonphotosensitive PI precursor (Beijing POME Technology Co., ZKPI-305) on silicate glass substrates (thickness, 0.5 mm). Subsequently, the samples were soft baked at 90°C for 3 min and 140°C for 30 min and hard baked at 200°C for 30 min and 300°C for 90 min to finish the imidization step. For preparing the CPI film, after spinning the coating adhesive of the CPI precursor, the sample was soft baked at 90°C for 3 min, followed by hard baked at a temperature that was risen in a gradient style (80°, 120°, 160°, 200°, 240°, and 290°C, each for 20 min). The film thickness was measured by a step profiler.

### Laser scanning process via a homemade platform

An experimental platform consists of an automated motion platform, a laser system, and an alignment system (shown in Supporting Information Text section A). An XeCl excimer laser (Coherent Inc., Compex205) with a wavelength of 308 nm, pulse duration of 25 ns, and a maximum repetition rate of 50 Hz was used as the laser light source. The optical transmission and shaping system can transform the original laser beam into a squared-shaped beam (2.8 mm by 2.8 mm) with uniform spatial energy distribution. The laser fluence and APN are two critical process parameters for the laser scanning process. The laser fluence was calculated by dividing the pulse energy by the beam area, and the pulse energy was measured by an energy meter (Coherent J-50MUV-248) from 100 pulses before each experiment. The APN is a nondimensional parameter that represents the number of irradiations that a unit area experiences during laser scanning.

### Mechanical peeling by a TRT tape

After laser scanning, a commercial TRT tape was attached to the surface of the PI film. The outer ring region of the sample should be irradiated by a high-fluence laser (>120 mJ/cm^2^) to make the initial peeling at the edge. Then, the PI film could be released from the transparent substrate with the help of external mechanical forces. The TRT tape was heated at 110°C to achieve a freestanding IWN-coated PI film.

### Characterization of interface foaming

For the measurement of the adhesion strength of the PI-glass structure, pull test equipment (model 5944, INSTRON) was used. As shown in fig. S9, the sample was bonded to a self-made aluminum block by a strong double-sided tape. The clamping force was set as ~100 N, and, after the force reached this level, the clamping stayed for at least 3 min. After the precompression, the sample was slowly pulled up at a constant peeling speed of 1.5 mm/s. The maximum tensile force was obtained from the force/displacement curve. For the measurement of the swelling of the PI film, a stainless-steel sheet with a circle hollow (diameter of 1 mm) was used as a mask, and the mask plate was affixed to the glass substrate. After scanning the entire hollow area with a fixed APN = 5, the optical phenomena were first photographed with an ultra–depth-of-field 3D microscope (DSX 510), and the swelling of the PI film in the irradiated area was measured by a step profiler (DektakXT). Then, the tested area can be scanned and measured again, until the PI film of the test area is separated from the substrate.

### Characterization of surface nanostructures

The morphology features of surface nanostructures were characterized by SEM (Helios NanoLab G3CX). To get a clear observation, all specimens were treated by depositing a thin film (~10 nm) of platinum by magnetron sputtering. To obtain side-view SEM images, a section of the sample was performed by focused-ion-beam. The analysis of SEM images was performed by Image J software. The detailed procedures are presented in Supporting Information Text section D.

### Characterization of multifunctional properties

A UV spectrometer (UH4150) was used to measure the transmission and reflectance spectra. The angle-resolved reflectance and transmittance spectra were recorded by an angle-resolved spectrometer. The wetting property of the film was investigated by WCA measurement. The bactericidal efficacy was measured by the cell-viability plate counts (the detailed method is presented in Supporting Information Text section E). The specific process of physical stability tests is presented in movie S2. The bacterial antiadhesive ability was examined by live/dead bacterial staining. Samples were incubated with *S. aureus* (10^7^ CFU/ml, strain ATCC 29213) at 37°C for 24 hours. After incubation, planktonic bacteria were gently washed away with phosphate-buffered saline and then stained with a SYTO 9/PI dye mixture for visualization and imaging using a confocal laser scanning microscopy. ImageJ was used to perform thresholding and bacterial surface coverage calculations.

### Simulation on gas-induced interface foaming

1D numerical simulations of the laser ablation and gas-induced interface foaming of the PI film were conducted by using the COMSOL and MATLAB software packages. The detailed model is presented in Supporting Information Text sections B and C.

### Preparation of insect wing–inspired CLs

A water transfer printing technique has been developed (see fig. S36 for details). A thin layer of PVA (with a mass fraction of 15%, ~10 μm thick, and cured at 110°C for 2 hours) was spin coated onto the ultrathin (~400 nm) PI film as a sacrificial layer to facilitate the release from the TRT tape after lase and peel. A hollow aluminum foil was used as a support to prevent the curling during the PVA dissolution. The aluminum foil was floated on the boiling water surface, and the PVA dissolved, obtaining the insect wing–inspired PI film floating on the water surface. Then, the receiving CL inside water retrieved the film from the water surface, and the film conformed to the CL with the assistance of the surface tension of the water.

### Eye-tracking test

The eye-tracking test was conducted by a commercial eye tracker (Pupil Core - Pupil Labs GmbH) along with its accompanying software (Pupil Capture). The interfering light source was the side lamp of a strong flashlight (T1000 - TCL Huarui Lighting Technology). The pupil center of the artificial eyeball moved in an approximately “∞” symbol path when the interfering light source was applied. The gaze points are shown in the images captured by the world camera of the eye tracker.

### Statistical analysis

The statistical analysis was carried out via GraphPad Prism 8.0, and the statistical significance of the difference (*P*) among groups was using unpaired Student’s *t* test. Data were presented as means ± SD (*n* ≥ 3). ***P < 0. 001; ****P < 0.0001.
