# Supplementary material for: Scalable manufacturing of multifunctional insect wing membrane via interfacial lase-and-peel strategy
Source: Sci Adv. 2025 Dec 10;11(50):eaea6934. doi: 10.1126/sciadv.aea6934 (PMC12694025; doi:10.1126/sciadv.aea6934)
Supplement: Supplementary file 1 — Supplementary text Figs. S1 to S36 Tables S1 to S4 Legends for movies S1 to S3 [file sciadv.aea6934_sm.pdf]

Science Advances
AAAS

Supplementary Materials for

Scalable manufacturing of multifunctional insect wing membrane via interfacial lase-and-peel strategy

Jing Bian *et al.*

Corresponding author: YongAn Huang, yahuang@hust.edu.cn

Sci. Adv. **11**, eaea6934 (2025)
DOI: 10.1126/sciadv.aea6934

The PDF file includes:

Supplementary text
Figs. S1 to S36
Tables S1 to S4
Legends for movies S1 to S3

Other Supplementary Material for this manuscript includes the following:

Movies S1 to S3

Supporting Information Text

Section A. Calculation of laser scanning efficiency

The laser scanning process is achieved by moving the sample with a motion platform (as shown in Figure S2). The motion platform moves at a constant speed v. Within the time interval between two adjacent pulses, the motion platform will generate a displacement $S = v/f$, where f is the laser pulse frequency, and the width and length of the laser spot are W_{beam} and L_{beam} respectively. At this time, the accumulated pulse number (APN) of the scanned area can be calculated by the following formula: APN = W_{beam}/S = $W_{beam} \times f/v$. The scanning area per unit time (i.e., processing efficiency) can be expressed as: $E_f = L_{beam} \times v$.

As illustrated in Figure S3, compared with our laboratory equipment, industrial laser lift-off systems significantly increase the laser spot size (e.g., 750 mm × 0.5 mm for the UVblade750HP laser system from Coherent Co. vs. 2.8 mm × 2.8 mm for our setup) and scanning frequency (600 Hz vs. 50 Hz), while maintaining higher spot uniformity (98% vs. 95%). When the scanning parameters are APN = 50 and laser fluence = 100 mJ/cm^2, the movement velocity of the motion platform of the industrial equipment v_i is 0.6 cm/s and the scanning efficiency reaches 45 cm^2/s. In contrast, for our experimental equipment, v_e is 0.28 cm/s and the scanning efficiency is only 0.0784 cm^2/s. Due to the fact that the laser fluence employed in our process is significantly lower than that required for laser lift-off, the actual power consumption in this instance is merely 225 W. By adjusting the scaling ratio of the cylindrical lens in the optical path of the laser equipment to triple the width of the laser spot, the laser power consumption rises to 675 W (reach full capacity), thereby enabling an increase in efficiency to 135 cm^2/s. Compared with our experimental equipment, industrial equipment demonstrates much better performance in terms of beam uniformity (1.8%, 2σ) and energy stability ($\sigma \leq 1\%$). Furthermore, the larger spot size of industrial equipment can eliminate the junctions between spots, further improving the uniformity of the scanning. Consequently, process scale-up primarily involves enhancing scanning efficiency (proportional to the beam size and the scanning frequency) without introducing additional risks.

Section B. Bulk photothermal model

During laser irradiation, the high temperature caused by the strong absorption of UV photons near the surface/interface leads to the bond-breaking within the PI. A bulk photothermal model was established to describe the bond-breaking of PI for the calculation of gas products. A heat equation is first given as (the coordinate is fixed at the PI−glass interface),

$$\frac{\partial T}{\partial t} = \frac{1}{\rho C_p(T)} \frac{\partial}{\partial z}\left[\kappa(T)\frac{\partial T}{\partial z}\right] + Q \tag{S1}$$

Here T is the temperature, ρ is the density, $C_p(T)$ is the specific heat, $k(T)$ is the thermal conductivity, and Q is the source term. The typical thermal depth $d_{th} = \sqrt{Dt_{\text{pulse}}}$ (D is thermal diffusivity and t_{pulse} is the pulse duration) and the optical penetration depth $d_{op} = 1/\alpha$ of the excimer laser are only ~100 nm, which are much smaller than the spot size, thus, the 1-D model is appropriate.

Typically, the thermal decomposition pathways of PI are complex, necessitating multi-step chemical kinetics models. However, in this study, due to the use of a very low-fluence laser (< 100 mJ/cm^2) and the fact that the temperature of PI does not reach the carbonization threshold, the photothermal model is specifically applied to describe the cleavage of the imine ring (the first step of PI thermal decomposition) in which the molecular chain undergoes reorganization and gas is generated. Due to the simple and determined reaction path, the decomposition of PI could be simplified as the single-step thermally activated reaction with a chemical kinetics equation for the fraction of broken bonds, n_b:

$$\frac{\partial n_b}{\partial t} = (1 - n_b)k_0\exp(-\frac{T_b}{T}) \tag{S2}$$

Here k_0 is a constant, and $T_b = E_b/k_B$ (k_B, Boltzmann constant) is the activation energy of the reaction.

The source term Q in Eq. S1 involves the absorption of the laser as well as the heat loss of chemical reactions,

$$Q = \frac{\alpha I}{C_p \rho} - \frac{L(1-n_b)k_0 \exp\left(-\frac{T_b}{T}\right)}{C_p \rho} \tag{S3}$$

Here $L = \triangle H_b N_0$ is the volumetric enthalpy for the reaction, $\triangle H_b$ is the enthalpy required to break one bond, and N_0 is the number of possible broken bonds per unit volume. The laser intensity I(z,t) is governed by the equation,

$$\frac{\partial I}{\partial z} = -\alpha I \tag{S4}$$

The absorption coefficient α is treated as a constant in consideration of a low degree of decomposition. The initial laser intensity at the interface was simplified as a rectangular square wave with a pulse width of 20 ns.

The broken bonds of PI will create gas products, which are mainly CO molecules cleaved from imide rings. The amount of gas products n_g is proportional to the total portion of broken bonds N_b:

$$n_g\ (\text{nmol}) = \frac{M_g}{M} = \frac{\rho}{M}\frac{m_g}{m_t}N_b = \frac{\rho}{M}\frac{m_g}{m_t}\int_0^{\infty} n_b(z)dz = 1.5 \times N_b\ (\text{nm}) \tag{S5}$$

Here, M_g is the mass of gas products per unit area, M is the molar mass of gas (28 g/mol for CO), ρ is the density of PI (~ 1.4 g/cm^3), m_g/m_t is the mass fraction that the decomposed PI have transformed into gas products (~ 30%, 4 CO for one monomer), and N_b is the integral of n_b along with the PI thickness. The detailed parameters used in simulations are listed in the Table. S1. The Eq. S1-S4 can be solved combined with the initial conditions ($T_{t=0} = T_{room}$, $n_{b,\, t=0} = 0$) and the boundary conditions ($T_{z\to\infty} = T_{room}$, $n_{b,\, z\to\infty} = 0$) via a commercial software, COMSOL. For sequential irradiations, considering a quite long pulse interval ($t_{interval} >$ 0.05 s), the temperature will return to room temperature, whereas the fraction of broken bonds inherits the calculation results of the previous irradiation. Finally, according to Eq. S5, the amount of gas can be estimated based on the calculation of the total broken bonds of interfacial PI.

Section C. Bubble nucleation & growth model

In this study, due to the extremely small scale, non-uniform gas concentration, laser-induced transient temperature changes, and the unclear physical properties of high-temperature polymer, accurately simulating the polymer foaming process faces challenges. Consequently, our research does not aim to simulate every detail of bubble nucleation, growth, and coalescence but instead focuses on establishing the relationship between laser parameters and the evolution of interface cavitation.

The simplified interface foaming process can be divided into three basic steps:

(1) Releasing gas products into the molten PI by laser irradiations.

(2) Nucleation of bubbles in the supersaturated solution.

(3) Growth of bubbles in a molten polymer.

The step 1 can be well described by the above bulk photothermal model. We proposed a model for steps 2 and 3 to deal with simultaneous bubble nucleation and bubble growth. For simplicity, the bubble nucleation model is derived from classical nucleation theory. The bubble growth model is derived from the classical bubble growth model which presents the rate of growth of a bubble in an oversaturated liquid-gas solution.

Despite being simplified, the bubble growth model which can effectively reflect the consumption of gas in the molten polymer caused by bubble growth (gas diffusion into the bubble driven by the concentration difference between the inside and outside of the bubble). It should be noted that our model does not take into account the actual bubble coalescence process because bubble coalescence occurs naturally and does not affect the evolution of interface cavitation. Since the bubble density no longer changes after the second stage, the subsequent bubble growth process reflects the expansion of interface cavities.

When estimating the gas concentration in the molten PI, we did not consider the gas diffusion process because there is a significant temperature gradient around the interface region, and diffusion coefficient will be significantly reduced far from the high-temperature region. To simplify the analysis, we focus to the 50-nm thick region with the highest temperature and assumed that the gas concentration is uniformly distributed within this region for bubble nucleation and growth simulation. It is assumed that nucleation occurs in the region of about 50-100 nm away from the PI-glass interface and only depends on the average concentration values of gas at time t, $C_g(t)$. The equation of the bubble nucleation rate $J(t)$ is based on the classical nucleation theory, as formulated by the following equation

$$J(t) = \left(\frac{2\gamma}{\frac{\pi M_w}{N_A}}\right)^{\frac{1}{2}} \exp\left(-\frac{16\pi\gamma^3}{3k_B T\left(\frac{C_g(t)}{k_H} - P_c\right)^2}\right) N_A \tag{S6}$$

where γ is the surface tension, M_w is the molecular weight of gas, N_A is Avogadro's number, k_B is Boltzmann's constant, T is the temperature, $\frac{C_g(t)}{k_H}$ is the saturation pressure, k_H is the solubility parameter, which is proportional to the reciprocal of Henry's constant, and P_c is the ambient pressure.

The average concentration of gas in polymer at time t, $C_g(t)$ is given by following equation:

$$C_g(t) = C_0 - \int_0^t \frac{4\pi}{3} R^3(t-t',t') \frac{P_D(t-t',t')}{R_g T} J(t')\mathrm{d}t' \tag{S7}$$

Where C_0 the concentration of gas resulted from the decomposition of PI, and the second term on the right of Eq. S7 is the consumption of gas due to the nucleation and growth of bubbles. $R(t', t-t')$ and $P_D(t', t-t')$ $= 2\gamma/R(t', t-t')$ represent the bubble radius and its approximate inside pressure when a bubble nucleated at t' and grew for a period of $t-t'$, respectively. The initial condition of the bubble that was nucleated at t' is considered as follows: $R(t',0) = R_0$, $P_D(t', 0) = 2\gamma/R_0$.

Once a bubble is nucleated, the surrounding gas molecule diffuses into the bubble through the gas-polymer interface. We perform a classic bubble growth model, in which the growth rate is simply relative to the concentration difference between the bubble boundary and the polymer solution $\triangle C = C_g - C_s$, the bubble radius, and the growth time. The rate of growth of a bubble $\mathrm{d}R(t', t-t')/\mathrm{d}t$ at time t in an oversaturated liquid-gas solution is given as:

$$\frac{\mathrm{d}R(t',t-t')}{\mathrm{d}t} = \frac{\kappa(C_g(t) - C_i(R(t',t-t')))}{\rho_\infty + 2\gamma/3R(t',t-t')}\left(\frac{1}{R(t,t-t')} + \frac{1}{(\pi\kappa(t-t'))^{1/2}}\right) \tag{S8}$$

Where κ is the coefficient of diffusivity of the gas in the liquid, $C_s = 2\gamma/(R(t',t\text{-}t')k_H)$ is the dissolved gas concentration for a saturated solution, which is considered as the gas concentration at the bubble boundary, ρ_∞ is the density of the gas under the ambient pressure, $\rho_\infty = M_w P_c/R_g T$.

During simulations, for a single irradiation, the bulk photothermal model first calculate the increment on C_g. Considering the termination of the bubble nucleation/growth due to the rapid temperature decrease (lowering the temperature below glass transition temperature) after the laser irradiation, the time for the bubble nucleation/growth is fixed at 300 ns for a single irradiation. For sequential irradiations, all the variables

inherit the calculation results of the previous irradiation. For simplicity, the temperature T and the material parameters is considered as fixed values. The equations are numerically integrated along time. The following Figure S5 shows the flowchart of the the bubble nucleation & growth simulation. It should be noted that we could not obtain actual material parameters due to the complexity of the material components (i.e., gas solubility, and rheological parameters of polymer at high temperatures). Therefore, all the parameters used in simulations refer to reported numerical studies on polymeric foaming process, which are listed in the Table S2.

Section D. Morphological analysis method

To obtain the specific distribution form, periodicity, radius, and height of the fabricated nanoarchitectures, we performed the analysis of SEM images with Image J software. An appropriate threshold was applied to keep the nanoarchitectures and reject the surface between them. The image was transformed to binary as shown in the following Figure S18. Finally, the function "analyse particles" was used to run statistics on the topside area S_{top} of nanoarchitectures. To exclude particles with radius below 10 nm (could be image noise), the overall outline fitting used to acquire the S_{top} was applied to particles with size of 300 nm^2 or higher. To roughly calculate the ratio of freestanding nanopillars, an additional filtering was applied to particles with the percentage of circularity to 50-100%.

The periodicity was also calculated from top-view SEM images. The measurements were first performed manually using image J software. The numerical density of the nanopillars were also measured through the above particles analysis. From the numerical density n (μm^{-2}), we can also estimate the average distance between two nanopillars according to the random radial distribution function. The average distance between two columns is written as: $d = n^{-1/2}$. This method can be used to perform cross validation of the manual measurements. The height was calculated from side-view SEM images. The measurements were performed manually using image J software. The statistical analysis was realized from a total number of 30 nanopillars.

Section E. Evaluation of bactericidal activity

The bactericidal activity of IWNs-coated PI film was quantitatively evaluated using a plate counting method. Specifically, bactericidal properties of the fabricated surfaces were studied by covering the IWNs-coated PI film (a smooth PI film as the control sample) on top of droplets of cell suspensions in rich medium, incubating, plating retrieved cells, and comparing the number of colonies that appeared. These bactericidal experiments were performed in humidity-controlled chambers, as shown in the following Figure S24.

Staphylococcus aureus (S. Aureus, strain ATCC 29213) was chosen because it is the most common bacteria in clinical infection. Besides, the surface of the dragonfly wing has been proved to be highly bactericidal against S. aureus Bacteria. Bacteria were cultured in rich media until mid-log phase. The cultures were then diluted into PBS to the concentration of 1×10^5 CFU/ml. Prior to the experiment, both the IWNs-coated PI film and control sample were autoclaved. A 24-well plate was used as a humidity-controlled experimental system to prevent evaporation. To assure even humidity, 2 ml of sterile water was added to periphery wells. 1 ml of cells and 1 ml of Luria−Bertani (LB) broth were added in the middle wells, and these wells were sealed with the PI films (8 mm × 8 mm) with the surface of IWNs facing down (for cell–material interactions). All experiments were performed at room temperature 37 °C to 24 h. Then, the test sample and coculture media in each well were transferred into a centrifuge tube and swirled fully. After that, 100 μL of the coculture media was taken out for 103-105 degrees of dilution and 100 μL of the diluents was used to coat on the Petri dishes with LB solid medium which were placed in the constant temperature incubator (37 °C) overnight (to obtain 30–300 colonies per plate). The antibacterial rates (R) for S. aureus were calculated based on the following formula: $R = (A - B)/A \times 100\%$, where A was the number of bacteria in the control group and B was the number of bacteria in the experimental group.

Section F. Effects of nanopillar profiles on its anti-reflection performance

We categorized the nanopillars into three types (as shown in Figure S27): high-density nanopillars (P_h), medium-density nanopillars (P_m), and low-density nanopillars (P_l). The test results indicate (see Figure S28a) that P_m exhibits the best anti-reflection performance in the visible light range (P_m is exactly the closest to nanopillars on the surface of a real dragonfly's wing). For the nanostructures with the size less than the wavelength, the light is insensitive to these nanostructures and tends to bend progressively, which is equivalent to passing through a homogeneous media with a gradient refractive index (RI). In general, there are two requirements should be followed to attain the maximum light transmittance for this kind of anti-reflective nanopatterns: 1) The height of protuberance structures has to be at least 40% of the value of the longest operational wavelength: $h = 0.4\lambda_2$; 2) The period of the structures has to be less than half the value of the shortest operational wavelength divided by the RI of the material: $\Lambda < \lambda_1/2n$; The period and height of P_m are both in accordance with the requirements, whereas the heights of P_h and P_l are slightly lower. Furthermore, as the clustering phenomenon of P_l is more prominent, it leads to an overly large actual period (between the clusters). We conducted FDTD simulations on three simplified models of nanopillars (Figure S28(b-d)). The results indicated that the interaction between light and the nanopillars of P_m is more prominent.

In addition to the aforementioned two parameters (period and height), the duty ratio of the nanostructures (e.g., the detailed profile from top to bottom) also plays an important role in reducing reflection. Based on the effective medium theory, as the profile changes, the RI of the equivalent media also changes. The nanostructures behave like a multilayer film with a continuously graded RI. As a consequence, some detailed features of our nanopillars play a critical role in enhancing anti-reflection performance. Our nanopillars feature a pedestal and an irregular height distribution (Figure S29a). We simplified the profile of our nanopillars for modeling. The root-like pedestal at the bottom is equivalent to a truncated cone structure, the part in the middle was approximately regarded as a cylinder, and the tip at the top was equivalent to a cone. The effective volume fraction f(z) can be calculated through the simplified profiles.

For the pedestal region:

$$f(z) = 1 - \frac{2\pi * \left(R_p * (1 - \frac{z}{H_p}) + r\right)^2}{\sqrt{3}d^2} \qquad (z < H) \tag{S9}$$

For the cylinder region considering the irregular height distribution:

$$f(z) = 1 - erfc\left(\frac{z-h}{\sqrt{2}\sigma}\right) \cdot \frac{\pi r^2}{\sqrt{3}d^2} \qquad (z < h - H_t) \tag{S10}$$

For the cone region:

$$f(z) = 1 - erfc\left(\frac{z-h}{\sqrt{2}\sigma}\right) \cdot \frac{\pi {r_t}^2}{\sqrt{3}d^2} \qquad (h - H_t \leq z) \tag{S11}$$
$$r_t = r - \frac{2}{3}(z - (h - H_t))$$

The effective refractive index n_{eff} at any elevation can be derived:

$$n_{eff}(z) = \sqrt{{n_c}^2 \bullet \frac{2(1-f(z))n_c^2 + (1+2f(z))n_a^2}{(2+f(z))n_c^2 + (1-f(z))n_a^2}} \tag{S12}$$

The irregular characteristics endow the nanostructures with a gradient profile, thereby forming a continuous RI change at the interface (Figure S29a). In contrast, traditional regular nanostructures still have RI discontinuities. We further verified this through FDTD simulations, and the comparison confirm that random height distributions and bottom pedestals can more effectively affect the electric field distribution to reduce reflection (Figure S29(c-d)).

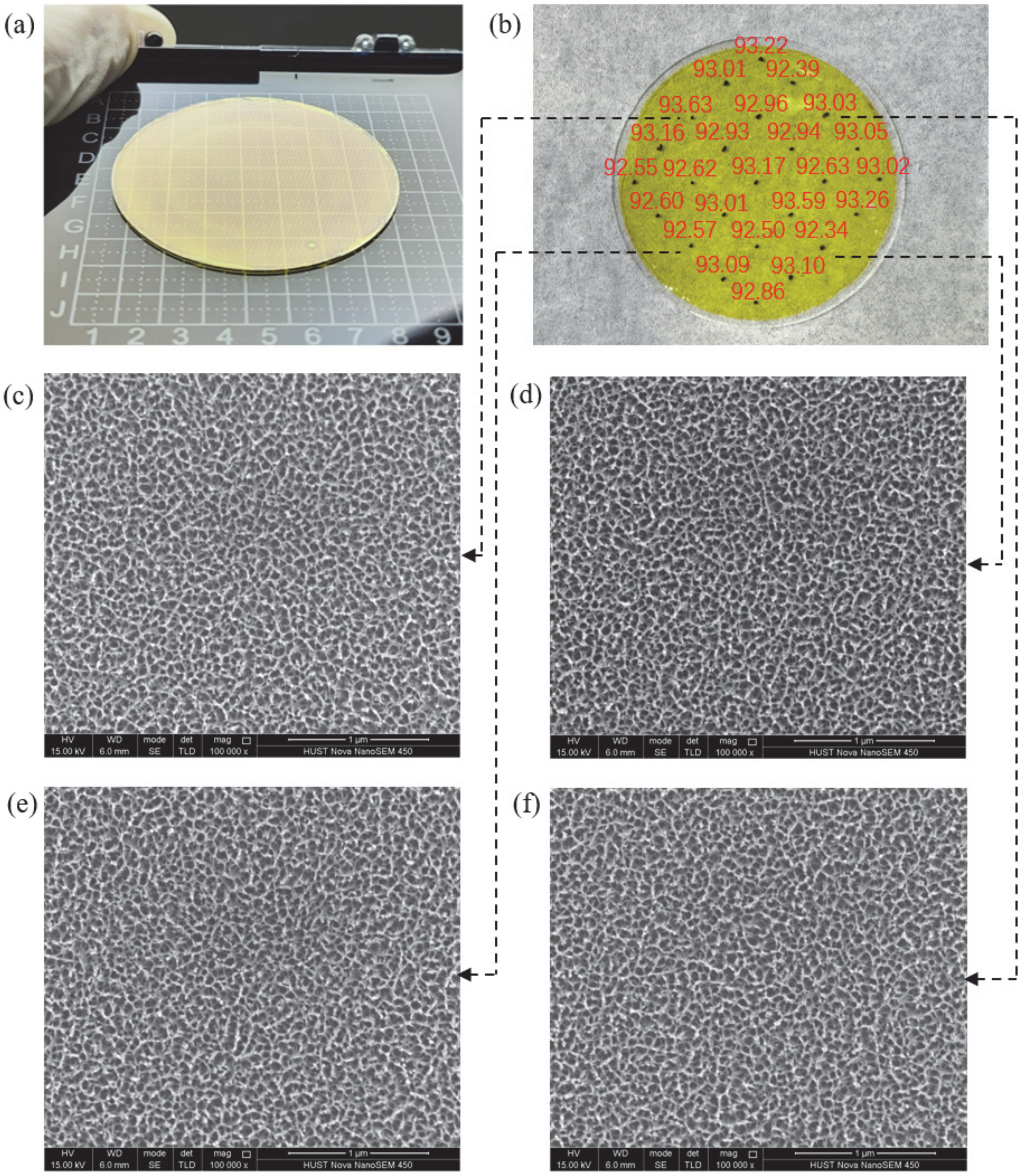

Figure S1. Demonstration of high spatial uniformity of the nanopillars over large-area. (a) A photograph of a large-sized sample after laser scanning reveals satisfactory uniformity. (b) Twenty-five regions were selected from the obtained nanostructured films for transmittance testing, and the average transmittance results of each region within the wavelength range of 550 - 800 nm were marked at the corresponding test positions. (c-f) SEM images acquired from four distinct locations confirm the morphological and density homogeneity of the nanopillars.

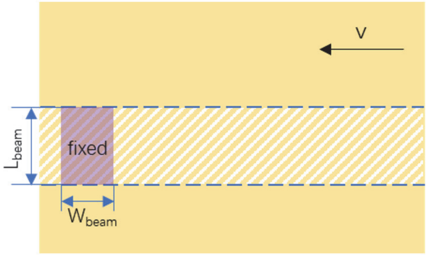

Figure S2. Schematic diagram of the excimer laser scanning process.

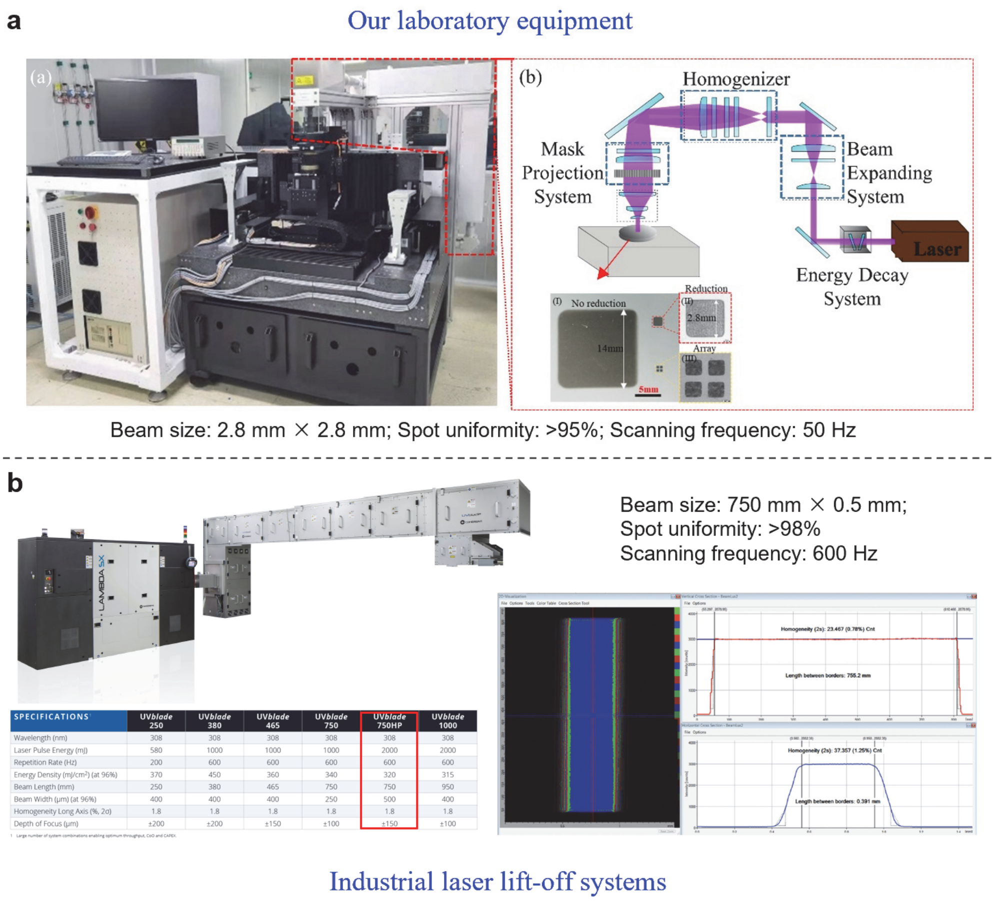

SPECIFICATIONS[1]	UV*blade* 250	UV*blade* 380	UV*blade* 465	UV*blade* 750	UV*blade* 750HP	UV*blade* 1000
Wavelength (nm)	308	308	308	308	308	308
Laser Pulse Energy (mJ)	580	1000	1000	1000	2000	2000
Repetition Rate (Hz)	200	600	600	600	600	600
Energy Density (mJ/cm^2) (at 96%)	370	450	360	340	320	315
Beam Length (mm)	250	380	465	750	750	950
Beam Width (μm) (at 96%)	400	400	400	250	500	400
Homogeneity Long Axis (%, 2σ)	1.8	1.8	1.8	1.8	1.8	1.8
Depth of Focus (μm)	±200	±200	±150	±100	±150	±100

1 Large number of system combinations enabling optimum throughput, CoO and CAPEX.

Figure S3. A comparison between industrial equipment and the equipment in our laboratory.

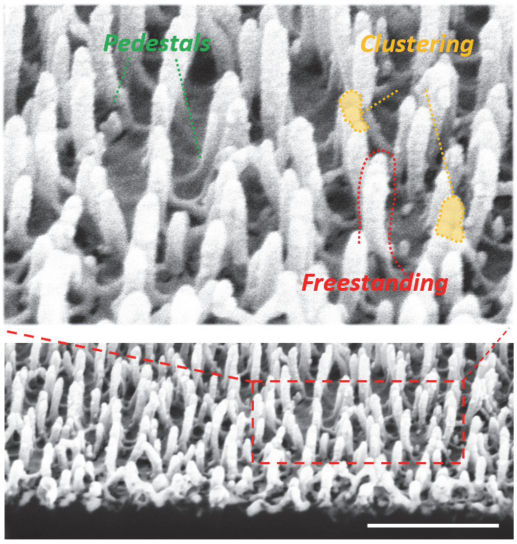

Figure S4. High-resolution side-view SEM image of artificial IWNs to show detailed features. Scale bar: 500 nm.

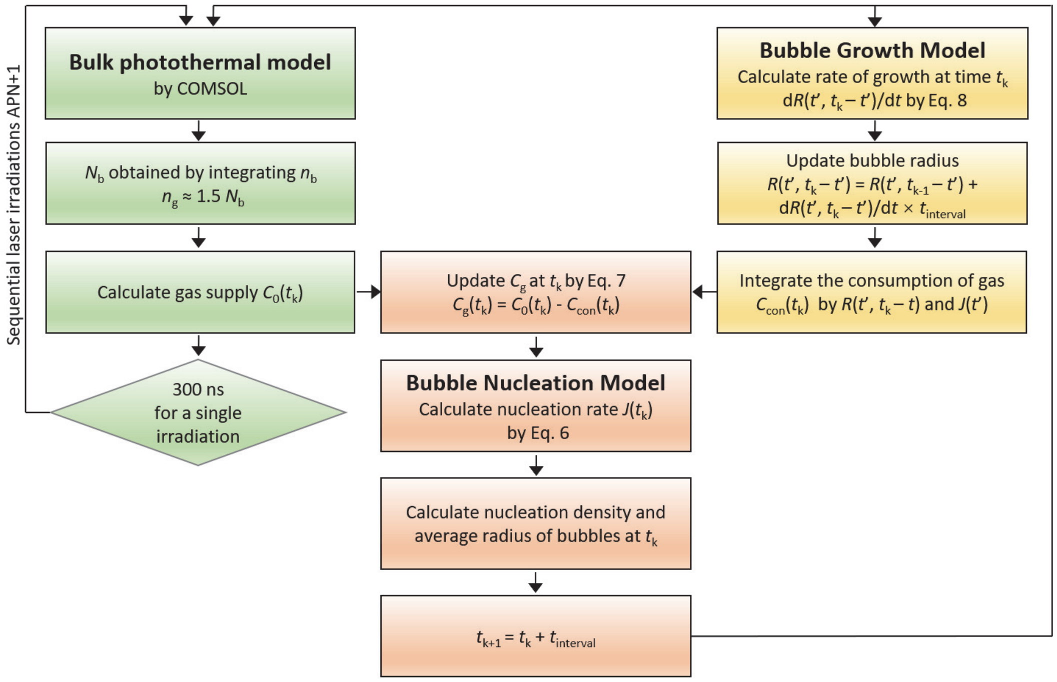

Figure S5. The flowchart of the calculation process of the models involved in Supporting Information Section C.

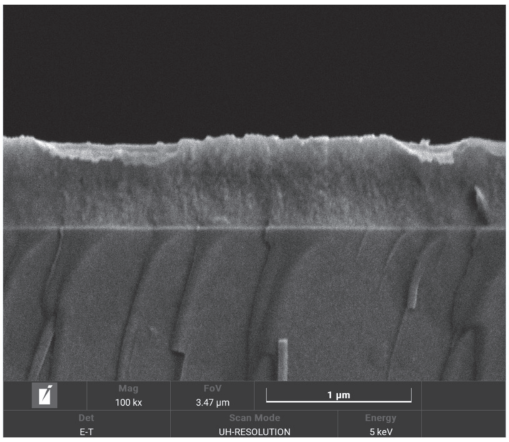

Figure S6. The microscopic morphology of the interface before irradiation, no cavitation could be found.

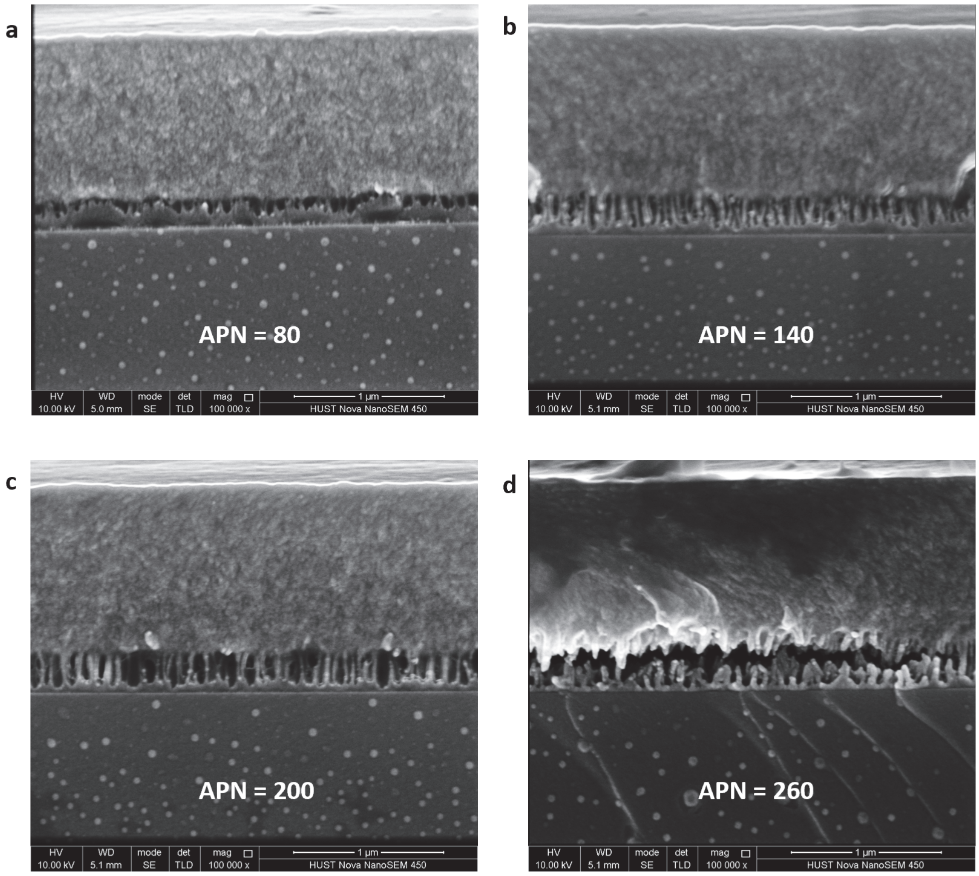

Figure S7. A series of cross-section SEM images of different stages of the interface foaming process. (a) bubble nucleation (85 mJ/cm^2, APN = 80), (b) bubble impingement (85 mJ/cm^2, APN = 140), (c) bubble wall rapture (85 mJ/cm^2, APN = 200), and (d) nanopillar formation (85 mJ/cm^2, APN = 260).

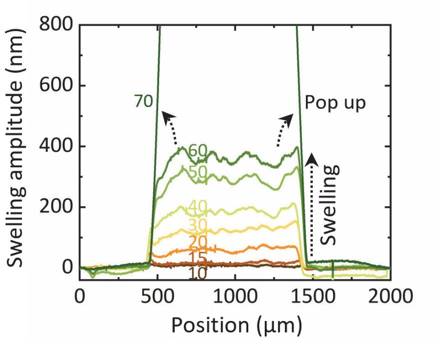

Figure S8. The measured surface profiles of the same region under increasing irradiation number. 100 mJ/cm^2, APN = 10-70.

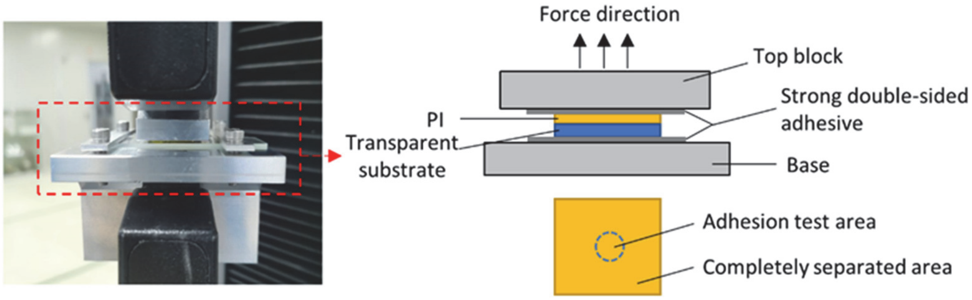

Figure S9. Schematic diagram of the experimental method of the interface adhesion test.

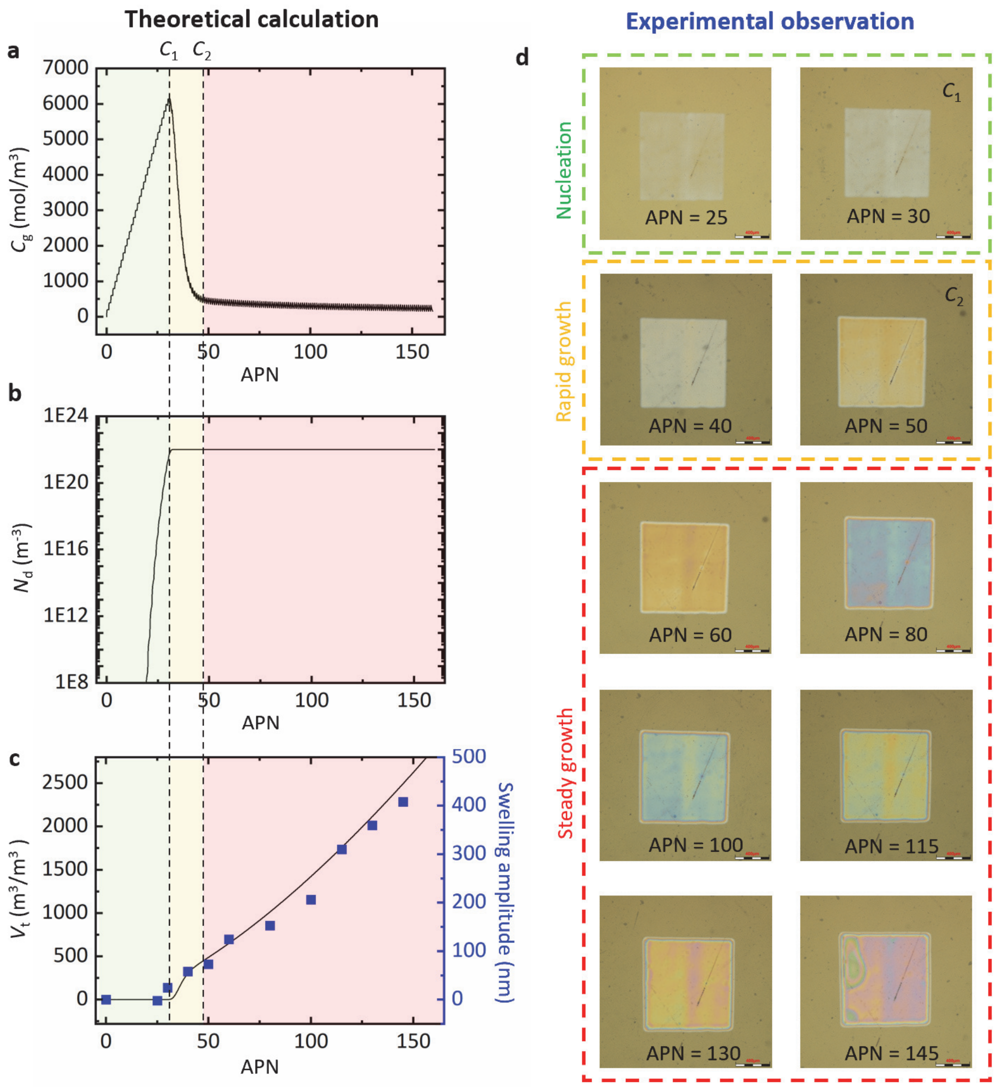

Figure S10. Experiments of the evolution of interface cavitation under the laser fluence of 95 mJ/cm^2. (a-c) The theoretical prediction of the gas concentration C_g, the bubble nucleation density D_d, and the total bubble volume V_t under 150 sequential irradiations. (d) Optical photographs of the same region under increasing irradiation number, APN = 0-150, the laser fluence was fixed at ~95 mJ/cm^2, the measured average swelling height is shown in the right of (c).

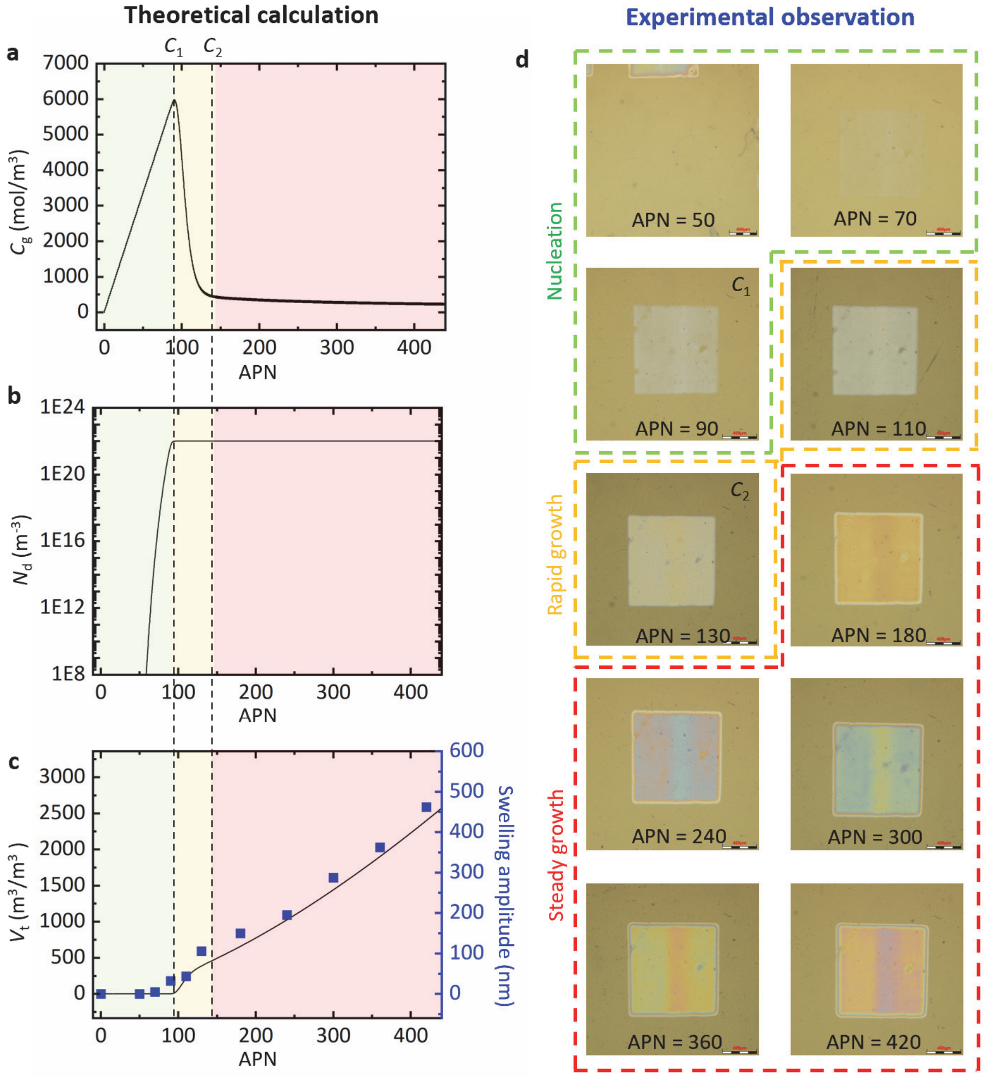

Figure S11. Experiments of the evolution of interface cavitation under the laser fluence of 85 mJ/cm^2. (a-c) The theoretical prediction of the gas concentration C_g, the bubble nucleation density D_d, and the total bubble volume V_t under 450 sequential irradiations. (d) Optical photographs of the same region under increasing irradiation number, APN = 0-450, the laser fluence was fixed at ~85 mJ/cm^2, the measured average swelling height is shown in the right of (c).

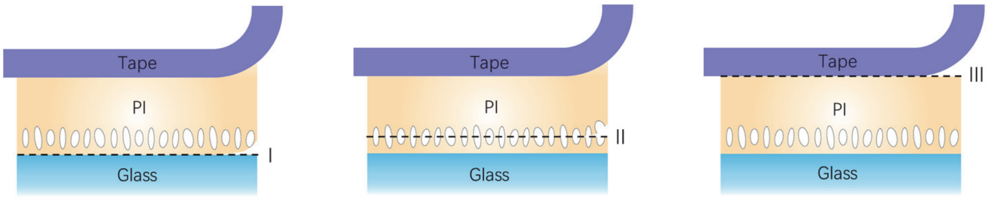

$G_{\text{I-c}} < [G_{\text{II-c}}, G_{\text{III-c}}]_{\min}$ $G_{\text{II-c}} < [G_{\text{III-c}}, G_{\text{III-c}}]_{\min}$ $G_{\text{III-c}} < [G_{\text{I-c}}, G_{\text{II-c}}]_{\min}$

Figure S12. Three possible separation interfaces when using a tape for mechanical peeling, only the interface II (inside the cavitation region) can produce IWNs.

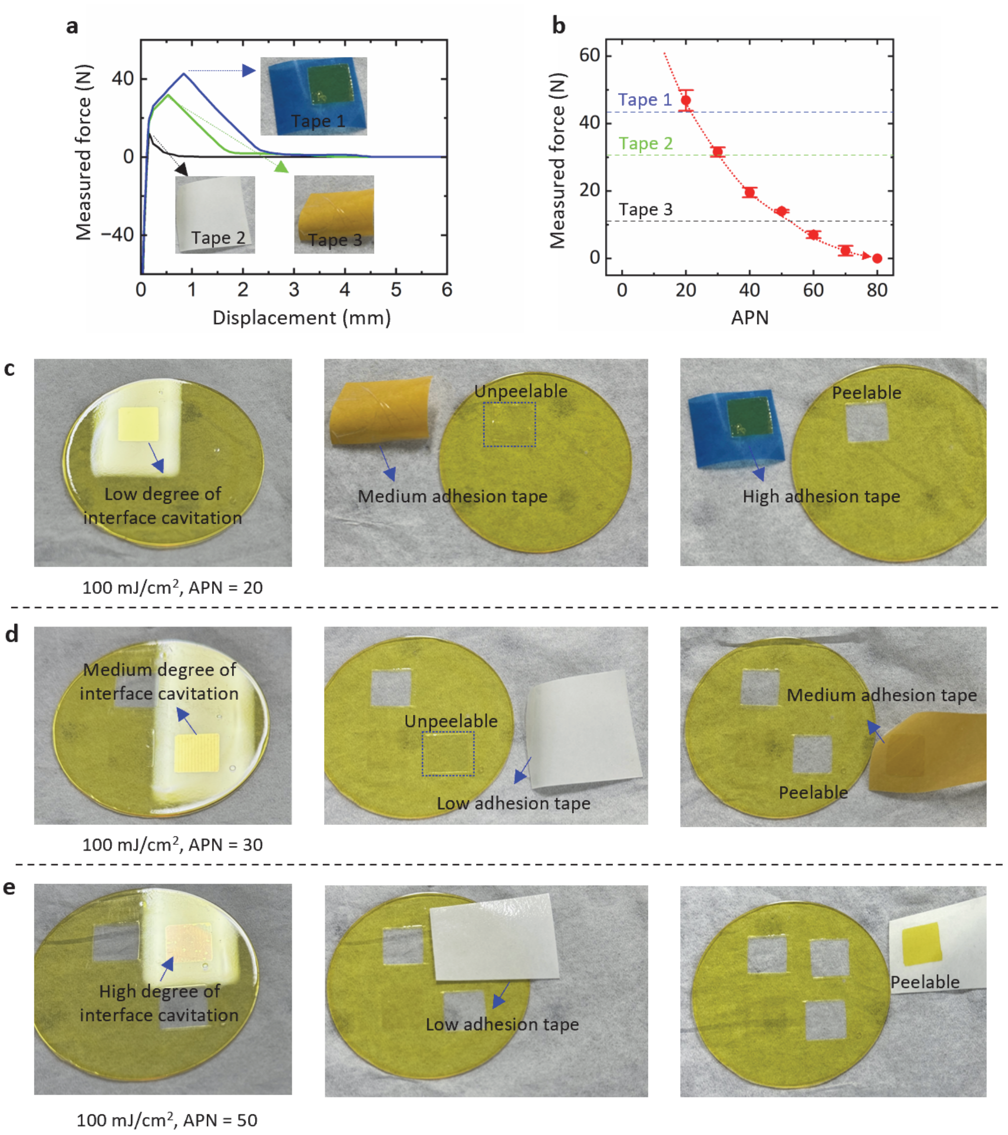

Figure S13. Mechanical peeling experiments using tapes of different viscosities. (a) The adhesion test results of three different adhesive tapes. (b) The adhesion force values were compared with the interfacial strength of PI-glass samples with different interface cavitation degrees. (c-d) The peeling experiments after laser-induced interfacial cavitation using three types of tapes with distinct adhesion.

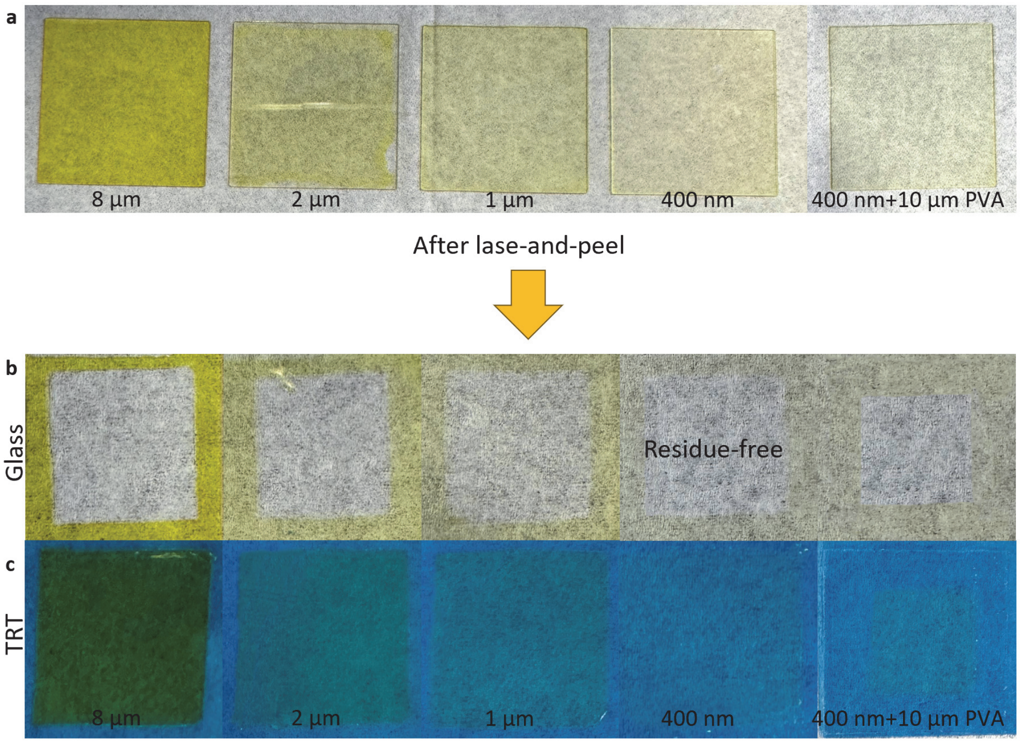

Figure S14. The photographs of PI film samples with varying thicknesses before (a) and after lase-and-peel (b-c), demonstrate that there is no residue on the surface of the glass substrate, and the PI films on the tape remain intact. This indicates that the thickness of the PI film does not influence the tape peeling step**.**

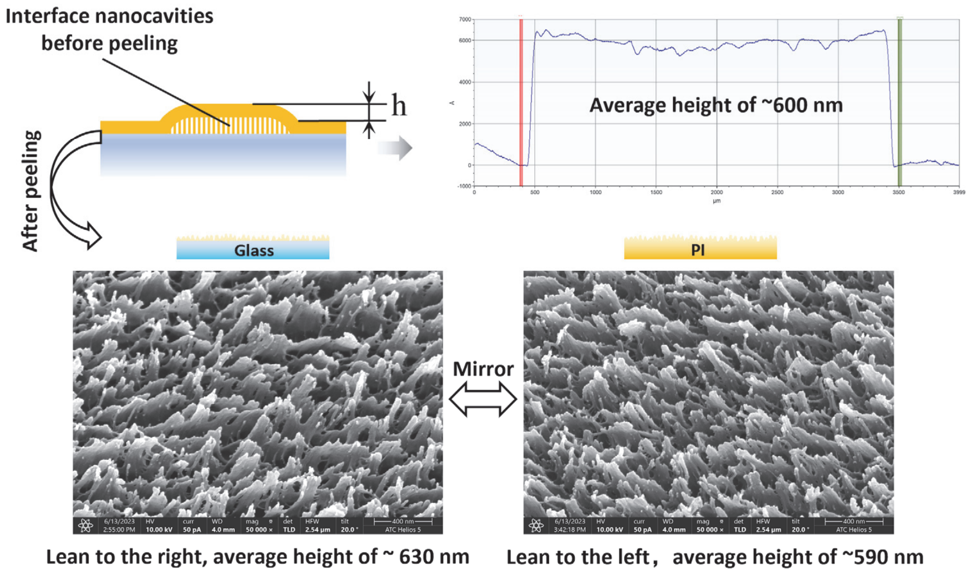

Figure S15. Comparison of the nanostructures on the peeled PI film and glass substrate from the exact same region after peeling. These nanostructures exhibit similar morphology features. Nanostructures on the glass slightly lean to the right, with an average height of ~ 630 nm, while nanostructures in the PI film lean to the left, with an average height of ~590 nm. The combined height of the nanostructures on the PI film surface and the glass surface exceeds that of the swelling amplitude (~ 600 nm) before peeling.

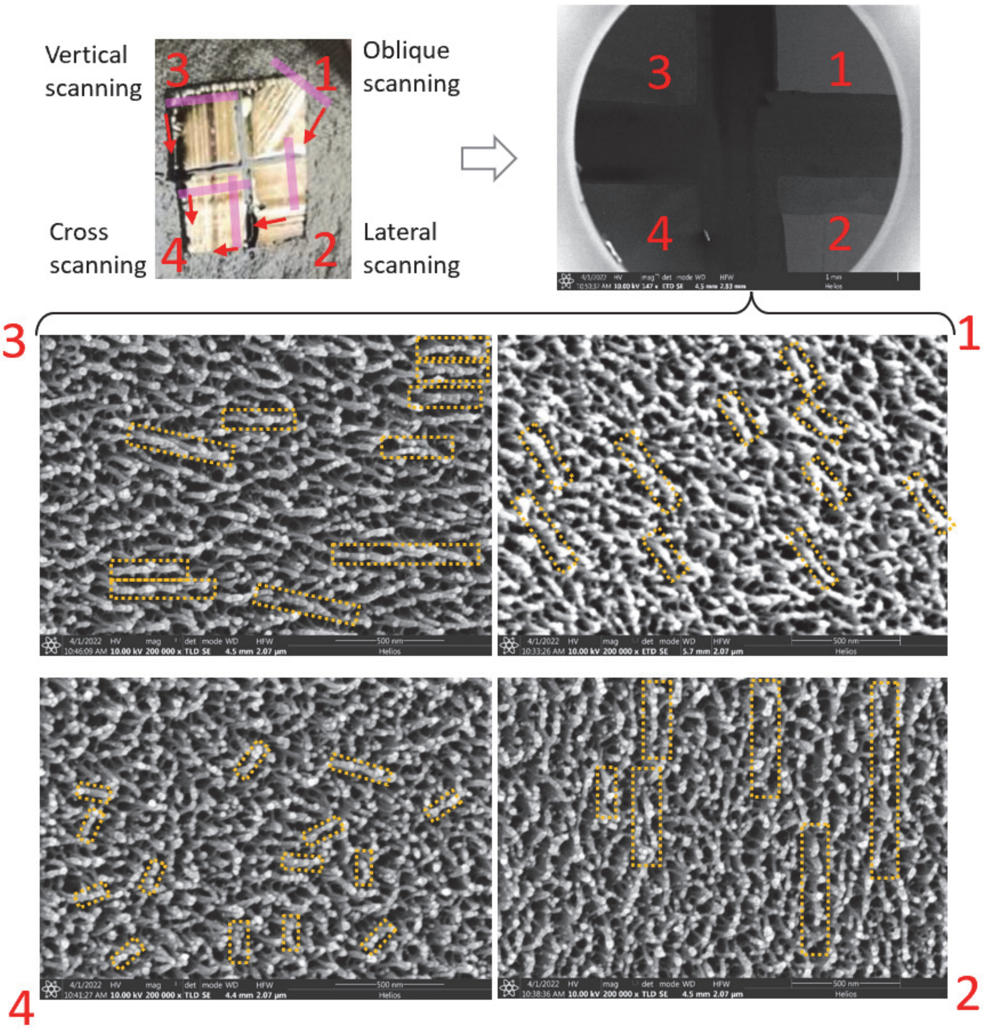

Figure S16. The morphology of nanopillars on the same sample varies with the direction of laser scanning. Laser fluence: 100 mJ/cm^2, APN = 40.

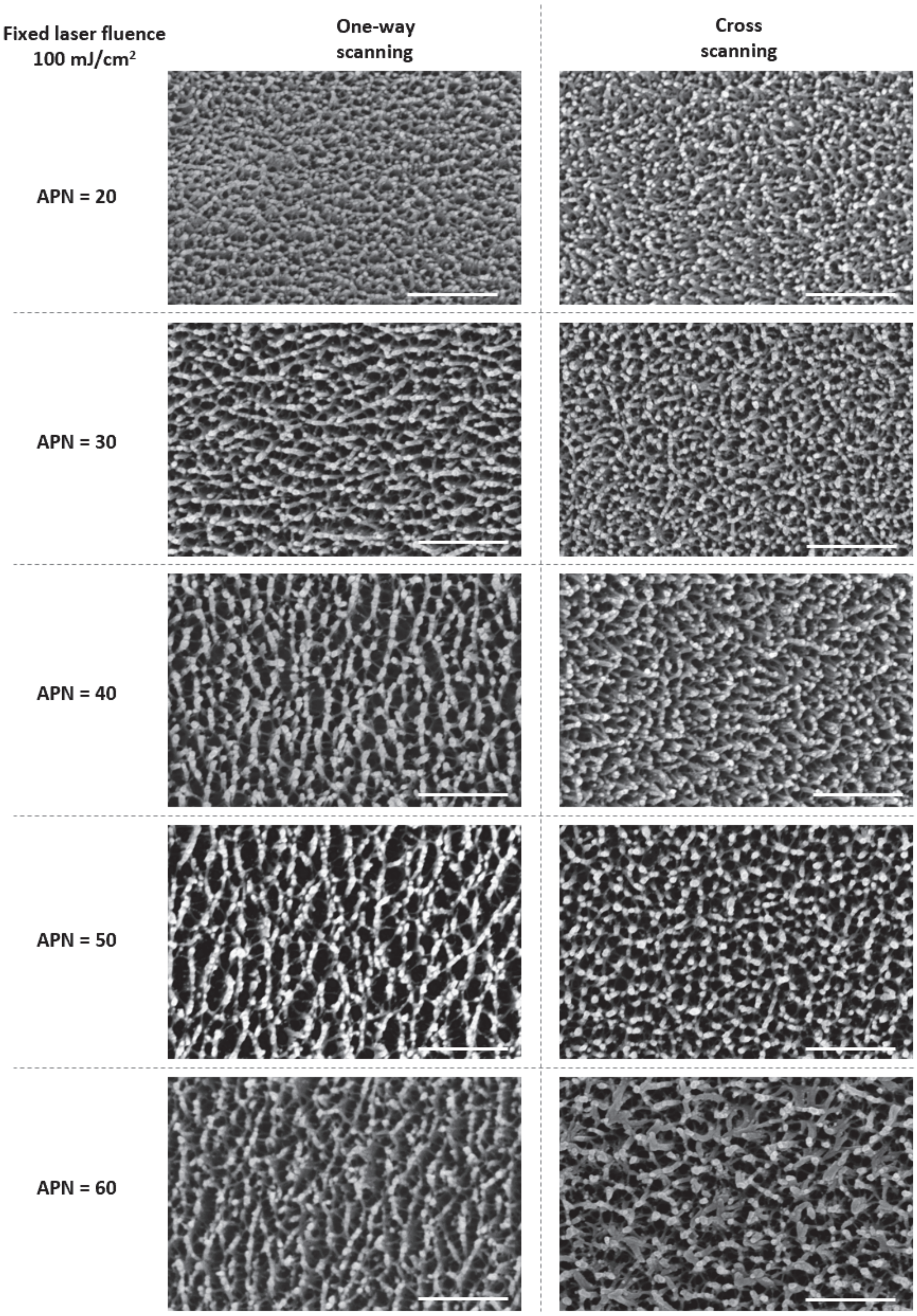

Figure S17. Representative SEM images of the surface nanostructures prepared by different scanning strategies and irradiation number (APN) at a fixed laser fluence of 100 mJ/cm^2.

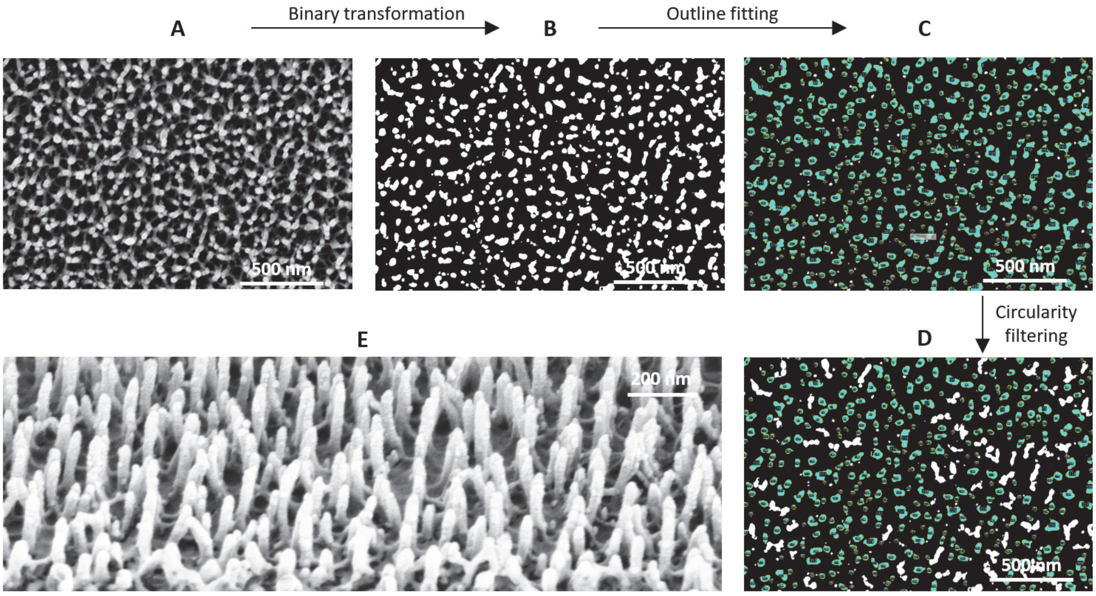

Figure S18. Morphological analysis method. (a) SEM image of APN = 50, F_l = 100 mJ/cm^2; (b) Binary transformation of image (a); (c) Outline fitting of image (b); (d) Circularity filtering of image (c); (e) A representative side-view SEM image for height measurements.

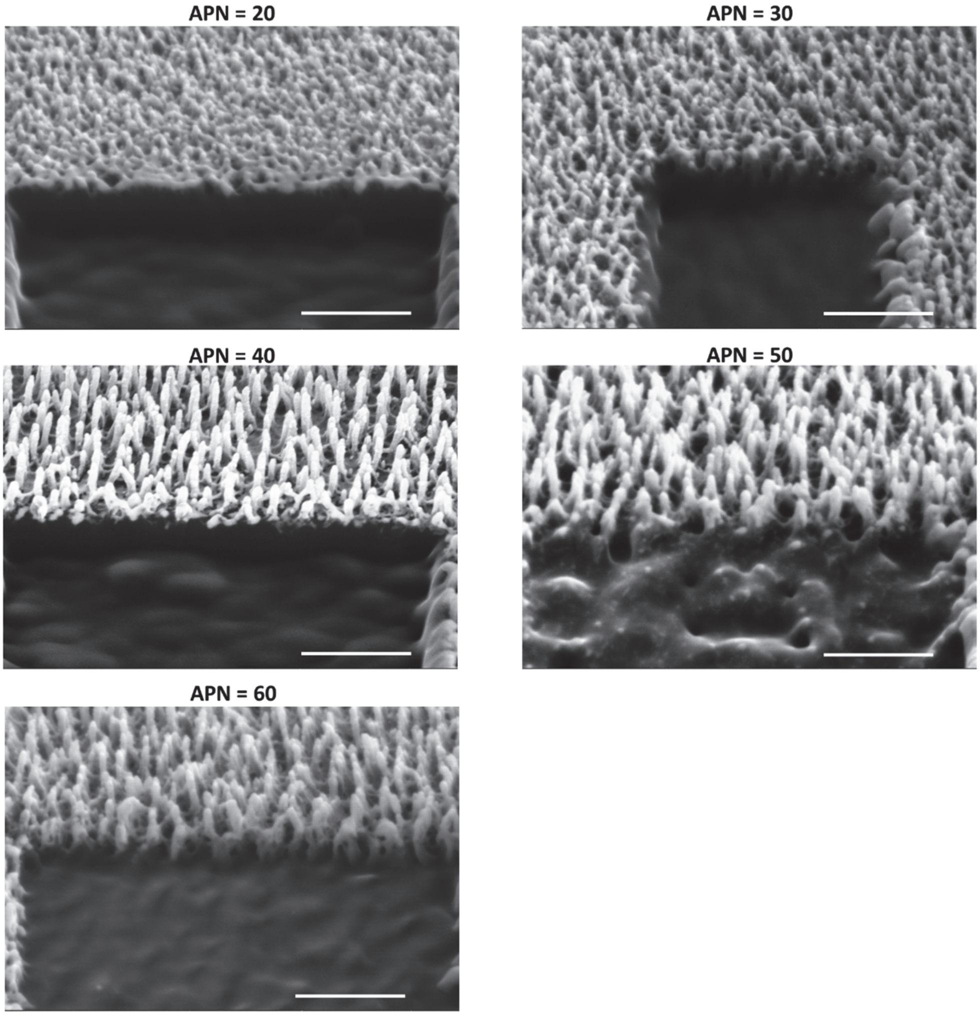

Figure S19. Representative side-view SEM images of the surface nanostructures prepared by different irradiation number (APN) at a fixed laser fluence of 100 mJ/cm^2. The results show the influence of the APN on the average height of nanopillars, which is the result of the growth of nanocavities along the vertical direction.

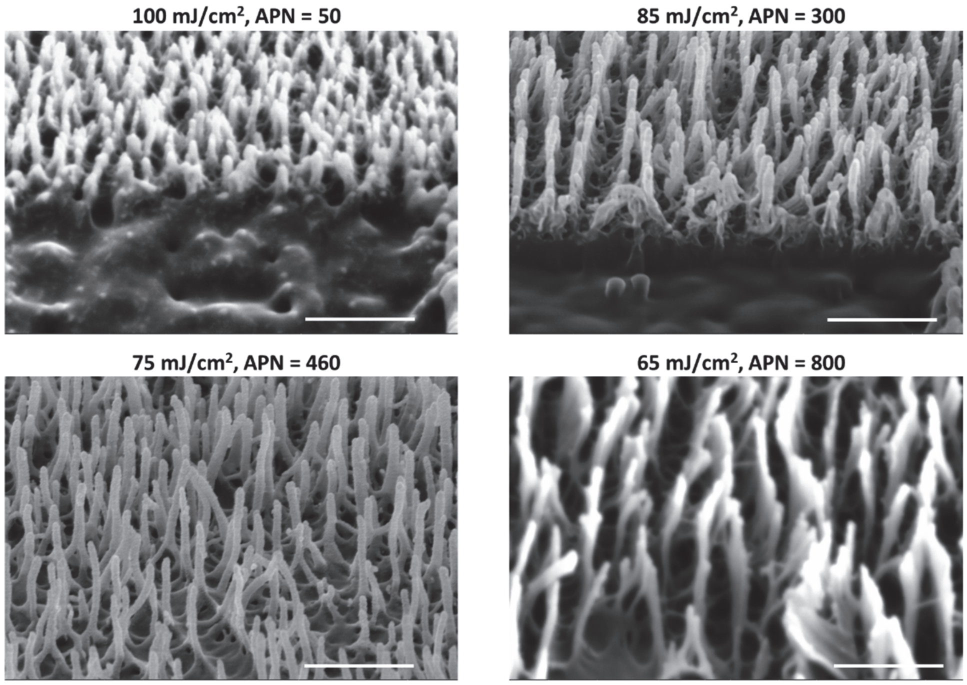

Figure S20. Representative side-view SEM images of the surface nanostructures prepared by different laser fluences. The obtained maximum height was found to be related to the applied laser fluence.

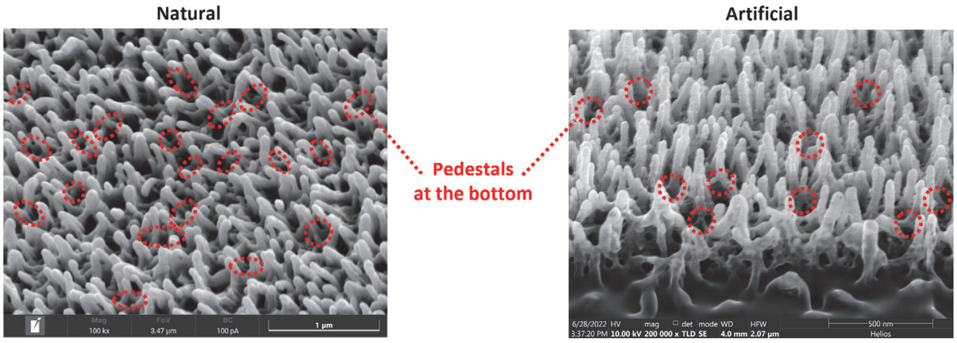

Figure S21. A detailed comparison of the nanostructures of natural dragonfly wings with artificial nanostructures. Both of them have pedestals at the bottom.

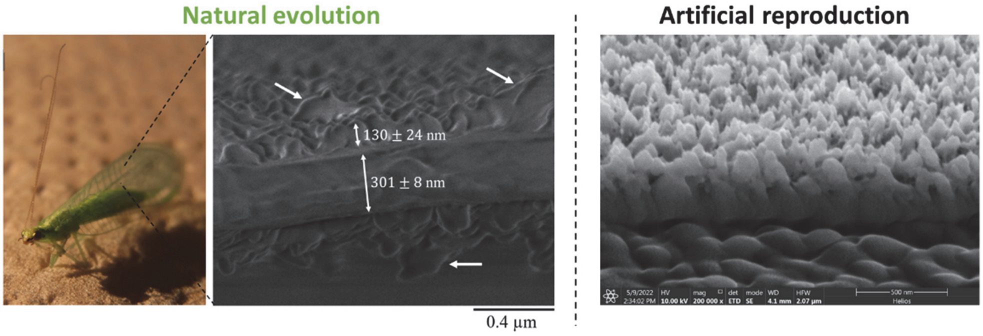

Figure S22. Comparison between the surface nanostructures (nanosheets) prepared by using one-way scanning and surface protrusions of natural green lacewing wings.

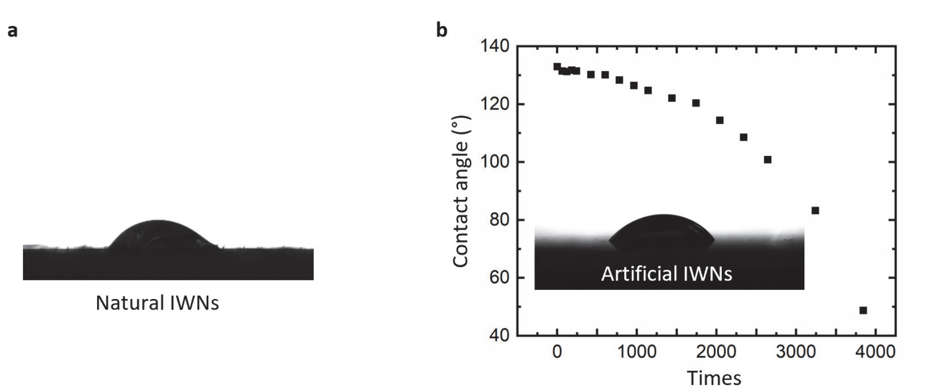

Figure S23. (a) Wettability of a single water droplet on the dragonfly wing surface after a long time. (b) Wettability dynamics of a single water droplet on our fabricated nanostructured surface for a long time.

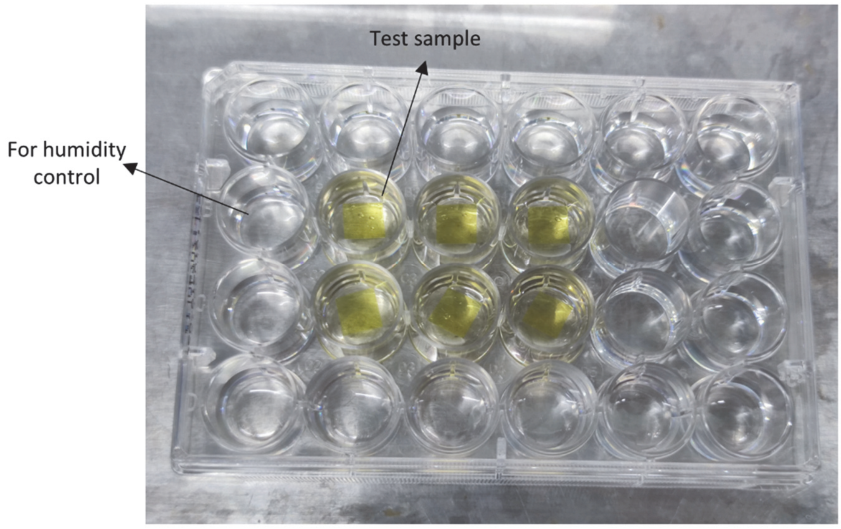

Figure S24. Evaluation of bactericidal activity. Bactericidal experiments were performed in humidity-controlled chambers.

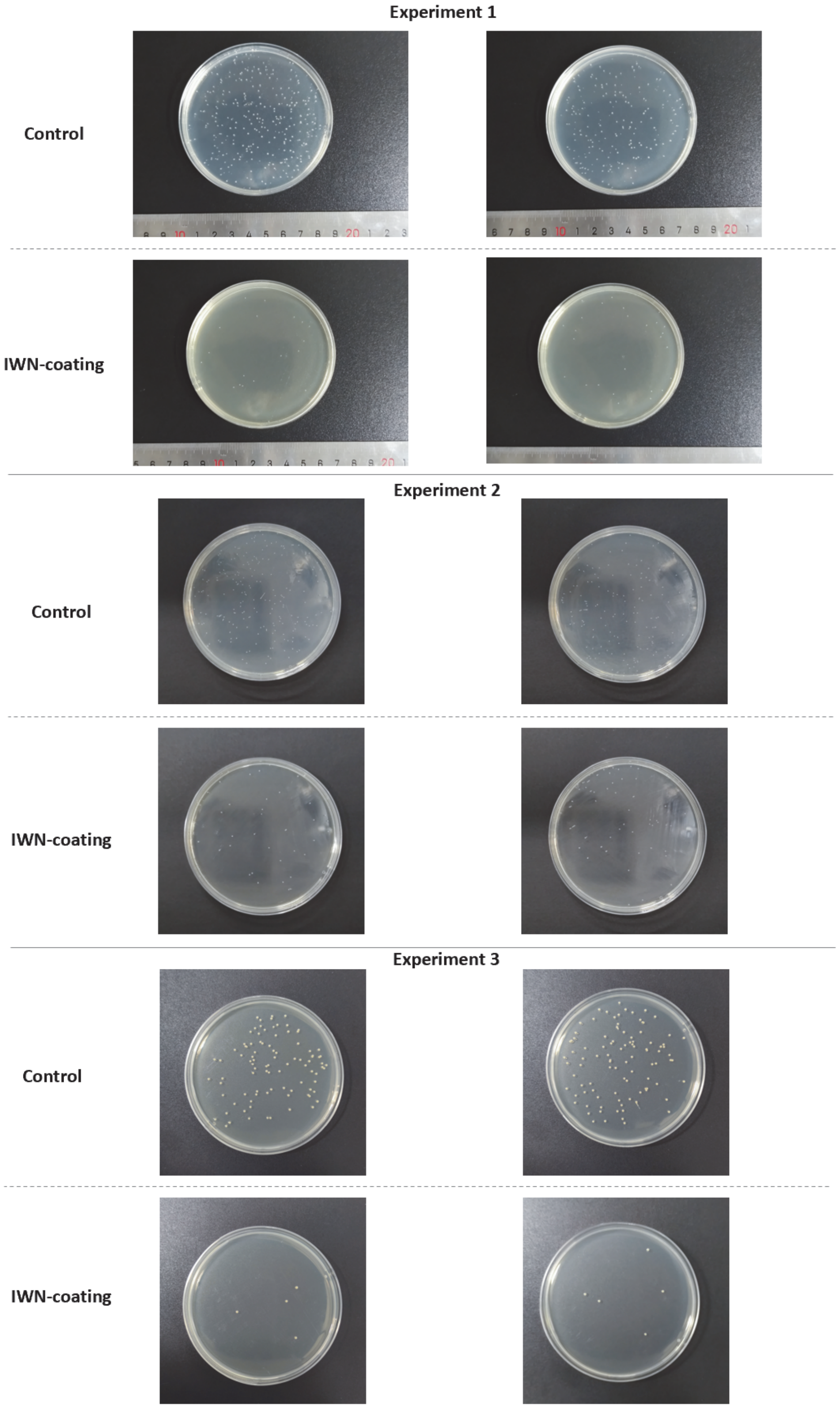

Figure S25. Results of plate counts for determining antibacterial rate of S. aureus of IWNs-coated PI films, compared with planar PI films as controls.

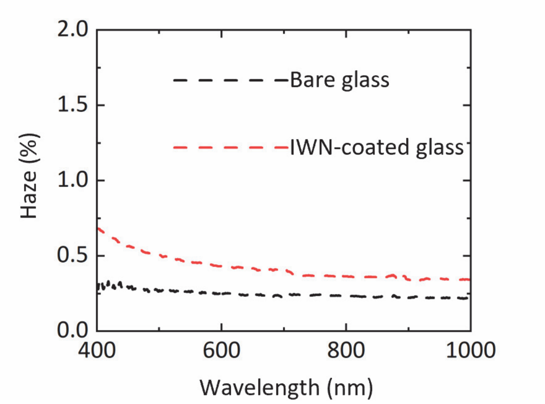

Figure S26. The test results for haze of an IWNs-coated silica glass and a bare silica glass. The IWNs-coating exhibits very low haze, demonstrating high clarity.

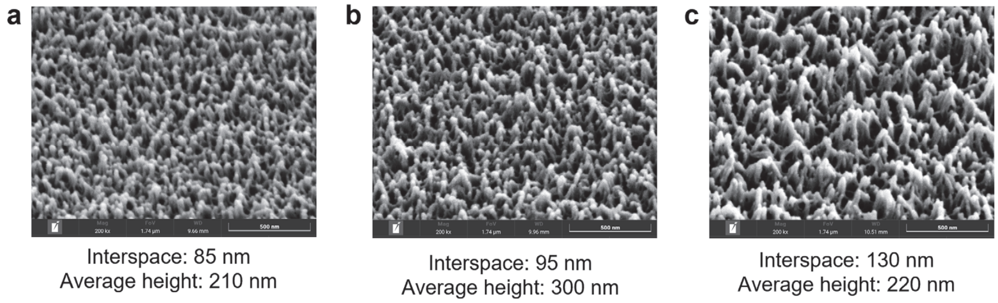

Figure S27. The surface morphologies of the three main types of nanopillars. (a) high-density nanopillars (95 mJ/cm^2, APN = 70); (b) medium-density nanopillars (95 mJ/cm^2, APN = 100), and (c) low-density nanopillars (95 mJ/cm^2, APN = 130).

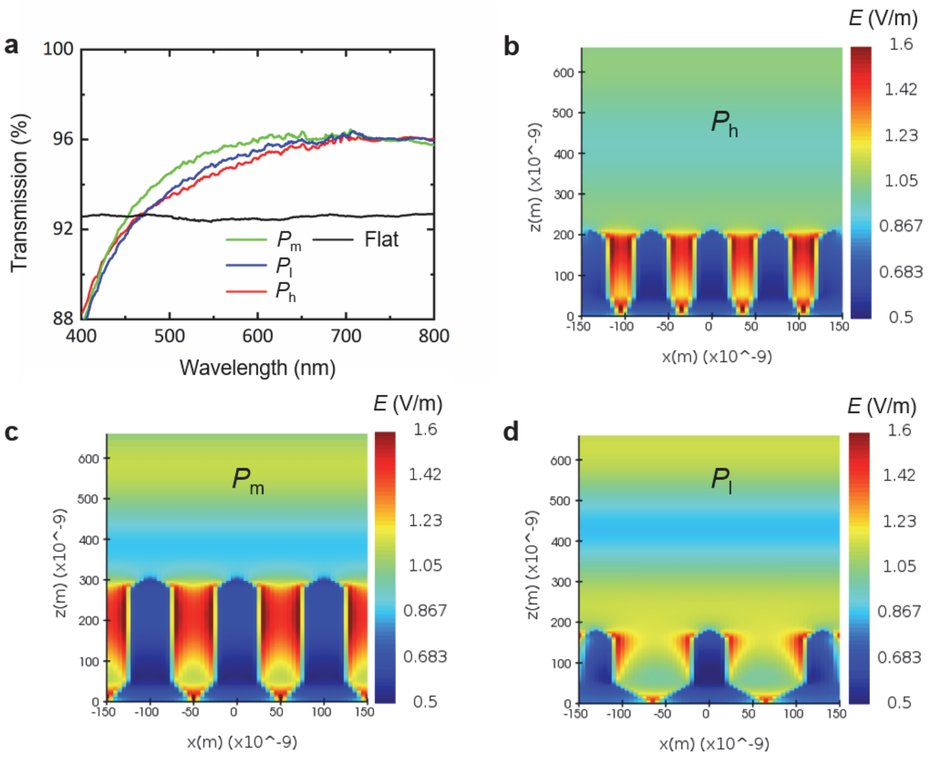

Figure S28. Effects of nanopillar profiles on its anti-reflection performance. (a) Test results of the antireflection performance of three types of nanopillars with distinct morphologies. (b-d) Finite difference time domain (FDTD) simulation of electric field (*E*) distribution at a wavelength of 700 nm under normal incidence for three simplified nanopillar models.

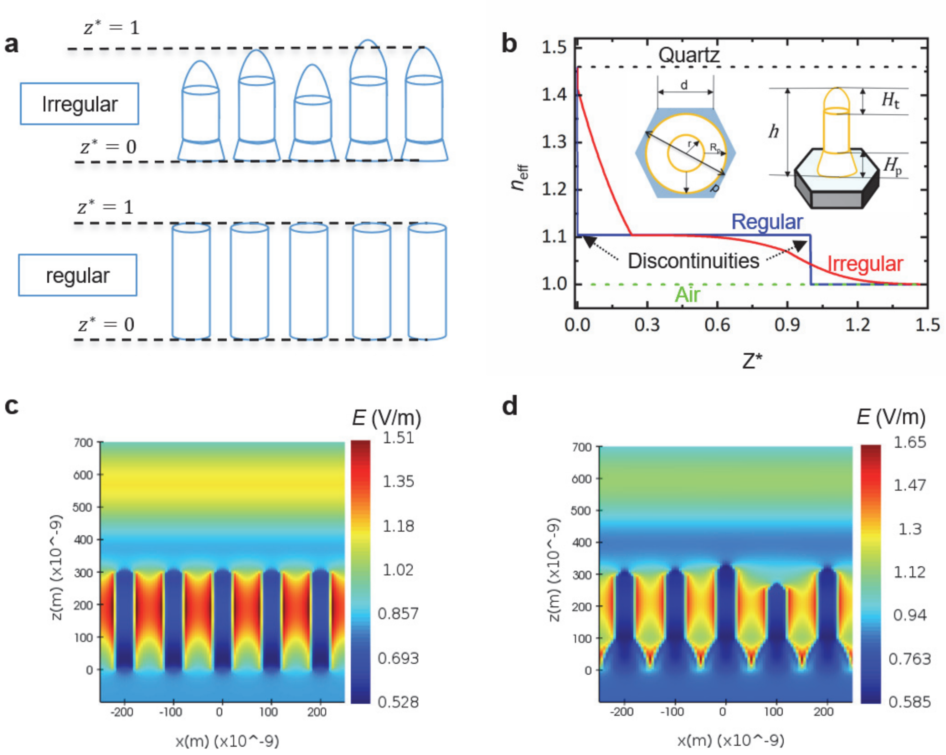

Figure S29. Effects of nanopillar profiles on its anti-reflection performance. (a) Morphological comparison between our irregular nanopillars and traditional regular nanopillars. (b) Comparison of the equivalent refractive index between our irregular nanopillars and traditional regular nanopillars. (c-d) FDTD simulations of electric field (E) distribution at a wavelength of 700 nm under normal incidence for our irregular nanopillars and traditional regular nanopillars. The inset schematic in (b) shows the structural parameters used for RI calculations and FDTD simulations. The average height h of the nanopillars is 300 nm, the standard deviation σ is 70 nm, the period d is 100 nm, the radius of the nanopillar r is 30 nm, the ring widths of the pedestal R_p is 20 nm, the height of the pedestal H_p is 100 nm, and the height of the top tip H_s is 30 nm. The RI of air n_a is 1, and RI of the substrate n_c is 1.46.

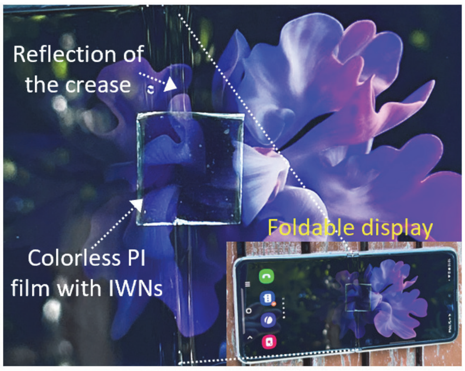

Figure S30. A foldable display coated with an IWNs-coated CPI film, the annoying reflection of the crease of the foldable display had been largely suppressed by covering an IWNs-coated CPI film.

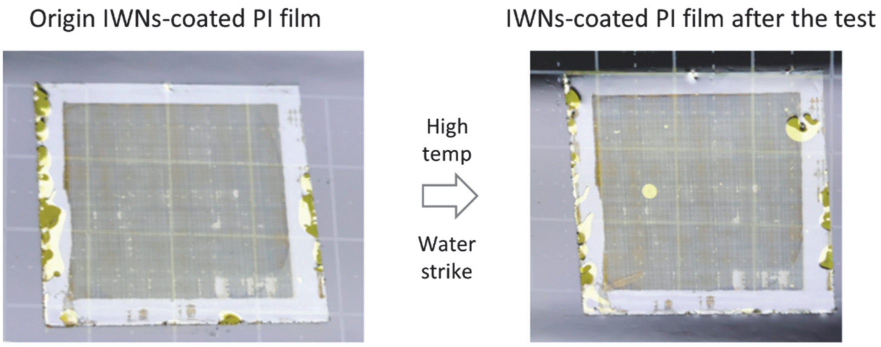

Figure S31. Optical images show a IWNs-coated PI film before and after the durability tests.

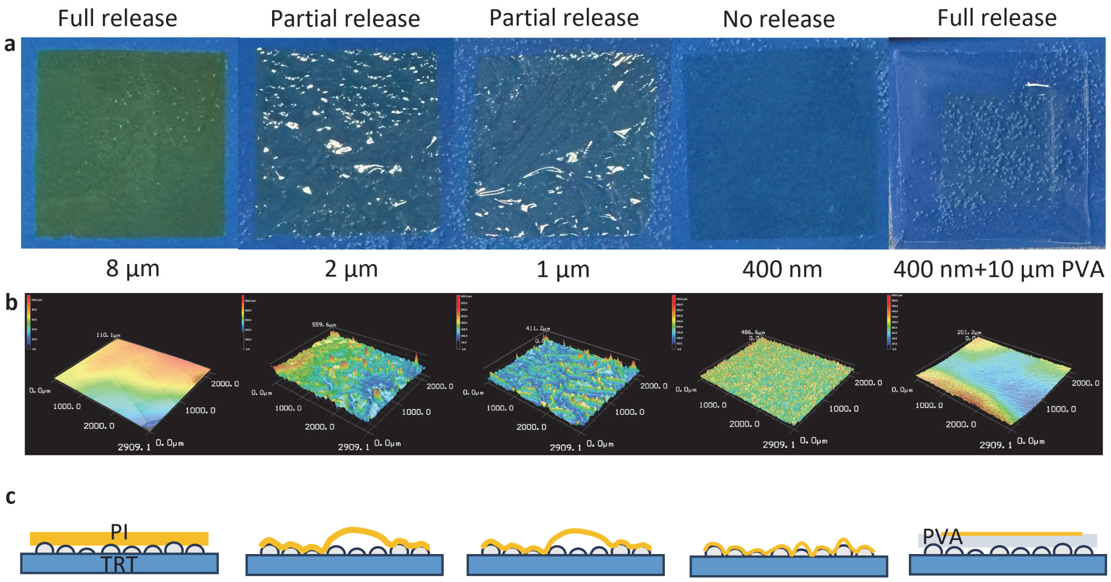

Figure S32. The photographs (a), surface profiles (b), and schematic illustrations (c) of PI film samples with various thicknesses after being released from the TRT. The results indicate the tape detachment process is highly sensitive to the thickness of the PI film, and it is difficult to achieve reliable release of films thinner than the sub-micron scale, while introducing a PVA sacrificial layer can achieve a complete release.

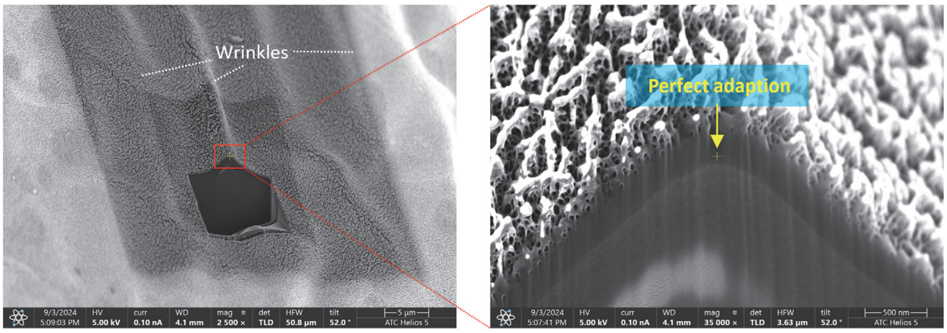

Figure S33. SEM observations show the exceptional conformability of the artificial wing membrane, which can adapt to surface wrinkles with a bending radius of less than 500 nm.

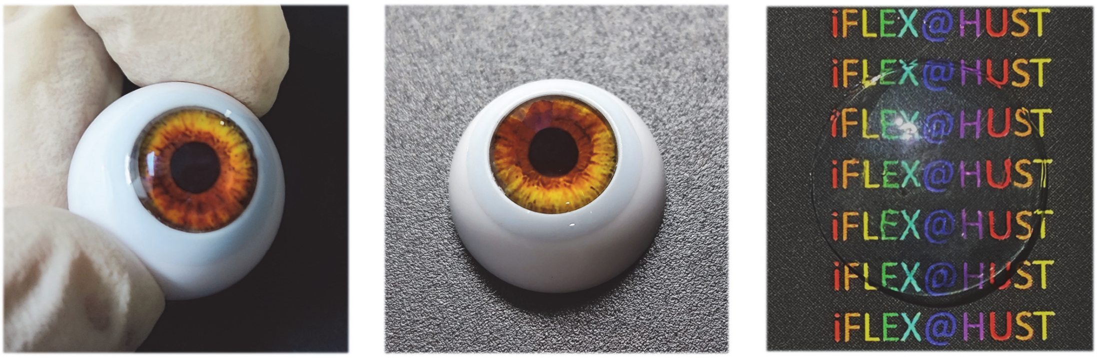

Figure S34. Photos of modified Contact Lenses. The artificial wing membrane is almost imperceptible.

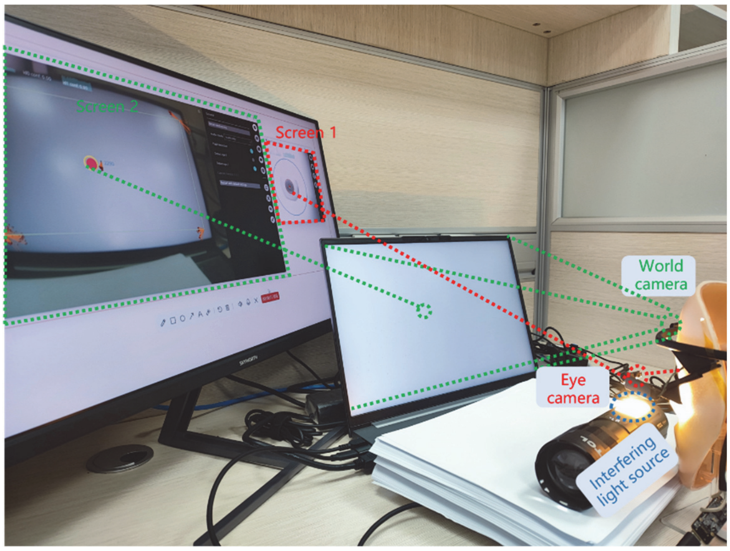

Figure S35. A photograph of the test scenarios for eye-tracking. A face model wears an eye tracker, using the monocular detection mode. The eye camera captures the image of the eyeball to track the pupil position (Screen 1), while the world camera captures the image of the environment in front of the eyeball model (Screen 2). The eyeball model moves, with the gaze point (i.e., the red point in screen 2) moving according to pupil positions tracked, to form the path of infinity symbol.

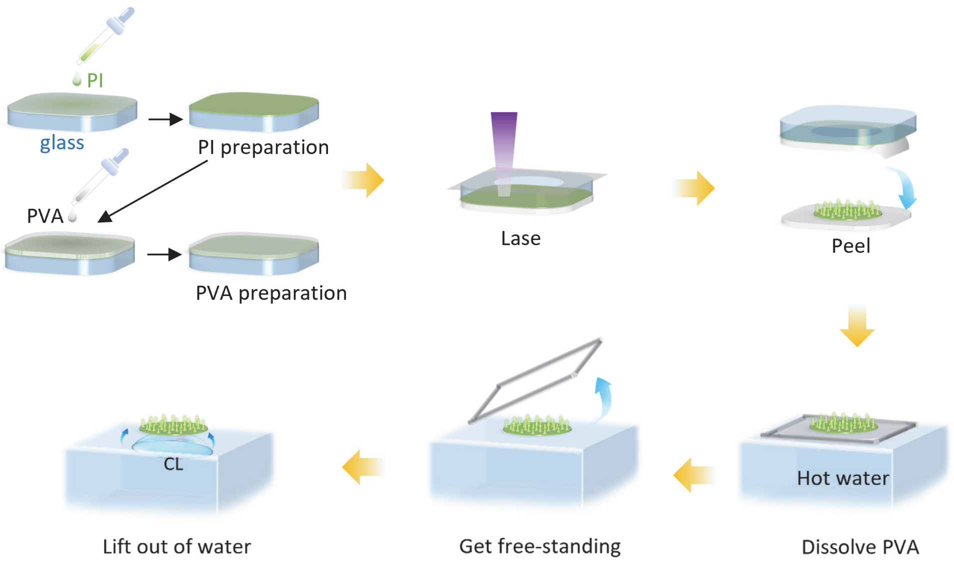

Figure S36. Schematic diagrams of the water transfer printing process to prepare insect-wing-inspired CLs. The key is to utilize a soluble adhesive (PVA) layer for the lase-and-peel process to obtain free-standing artificial wing membrane

Table S1. Parameters used in calculating the amount of gas by the bulk photothermal model

Parameter	Value
Activation energy T_a [K]	23140
Pre-exponential factor k_0	2.67×10^{12}
Volumetric reaction Enthalpy L [J/cm^3]	From 6500 ~ 9750
Reaction enthalpy per bond $\triangle H_b$ [eV]	1.4 ~ 2.2 eV
Number density of bonds N_0	2.71×10^{22}
Density of PI ρ [g/cm^{-3}]	1.42
Density of glass ρ [g/cm^{-3}]	2.37
Specific heat of PI C_p [J/g·K]	$2.55\text{-}1.59 \times \exp[(T_0\text{-}T)/460]$
Specific heat of glass C_p [J/g·K]	$1.53\text{-}0.79 \times \exp[(T_0\text{-}T)/638]$
Thermal conductivity of PI κ [W/cm·K]	$1.55 \times 10^{-3}(T/T_0)^{0.28}$
Thermal conductivity of glass κ [W/cm·K]	$7.9 \times 10^{-4}T^{0.43}$
Absorption coefficient α [cm/s]	65000

Table S2. Parameters used in calculations of the polymer foaming process by the bubble nucleation & growth model

Parameter	Value
Surface tension γ [N/m]	0.03
Molecular weight of gas M_w [g/mol]	28
Temperature T [K]	1100 ~ 1600
Henry's constant k_H [mol/m^3·Pa]	20e-05
Initial radius of the bubble R_0 [nm]	3
Coefficient of diffusivity κ [m^2/s]	2e-09
Ambient pressure P_c [Pa]	1.013e5

Table S3. Comparison of manufacturing techniques for biomimetic nanostructures.

Method	Process speed	Process simplicity	Size compatibility	Manufacturing cost	Aspect ratio	Morphological regulation	Reference
Reactive ion etching	Medium (20 minutes for up to 300 cm^2)	Low (7 steps)	Medium Limited by etching equipment (~300 cm^2)	Cr, PS nanospheres	~3.2	Yes (Varying etching time)	[27]
Metal-assisted chemical etching	High (3-6 minutes for up to 1400 cm^2)	Medium (5 steps)	Medium Limited by E-beam evaporator (~1400 cm^2)	SiO_2, Ni, Au	/	Yes (Varying initial thickness of the Ni film and annealing temperature)	[28]
Template assistance	Low (30 minutes for 16 cm^2)	Medium (4 steps)	Low Limited by template (16 cm^2)	Anodic aluminum oxide templates	~2.0	No (Diverse templates needed)	[23]
Nanoimprint	Medium (1 minute for 30 cm^2)	Medium (4 steps)	Low Limited by mold (30 cm^2)	Glassy carbon molds	~3.1	No (Diverse molds needed)	[24]
Ultrafast laser processing	Medium (A few minutes for 25 cm^2)	High (1 steps)	High	/	~1.3	Programmable (Varying laser fluence, pulse number)	[13]
Lase-and-Peel	Very high (> 100 cm^2/s)	High (2 steps)	High	TRT Tapes (No expensive cost)	~4.4	Programmable (Varying laser fluence and pulse number)	This work

Table S4. The dimensions of the natural IWNs found in the literature and the artificial IWNs produced in this study.

Insect species	Height (nm)	Interspace (nm)	Diameter/Structure width (nm)	Structural similarity	Detailed characteristic	Reference
Dragonfly	241	123	53	90% for Sample-2	Irregular array of single and clustering nanopillars	[15]
Planthopper	520	132	47	92% for Sample-4	Randomly oriented nanopillars	[19]
Glasswing butterfly	500	120	100	82% for Sample-4	Irregularly arranged, random height and width distribution, cone shaped pedestal	[12]
Damselfly	433	116	48	96% for Sample-3	Irregular array of single and clustering nanopillars	[18]
Cicada	200	117	90	81% for Sample-2	Regular array of spherically capped cones	[51]
Lacewing	950	300	80	72% for Sample-5	Interconnected netting composed of ridges	[17]
Sample-1 100 mJ/cm^2, APN = 50	205	99	43		Irregularly arranged, random height and width distribution, tree-root-like pedestal	This work
Sample-2 100 mJ/cm^2, APN = 60	193	128	50			This work
Sample-3 85 mJ/cm^2, APN = 300	438	115	52			This work
Sample-4 75 mJ/cm^2, APN = 460	498	136	57			This work
Sample-5 65 mJ/cm^2, APN =800	700	200	60			This work

Structural similarity is defined as the arithmetic mean of the three relative ratios of the basic sizes.

Movie S1. The whole fabrication process of artificial IWNs via Interfacial Lase-and-Peel Strategy.

Movie S2. The testing process of the mechanical stability of natural/artificial IWNs.

Movie S3. The infinity symbol calligraphed by eye movement under strong light interference using plain/modified CLs.
